# Application of the network scale‐up method to estimate the sizes of key populations for HIV in Singapore using online surveys

**DOI:** 10.1002/jia2.25973

**Published:** 2023-03-15

**Authors:** Sharon Esi Duoduwa Quaye, Yuwei Cheng, Rayner Kay Jin Tan, Joel R. Koo, Kiesha Prem, Alvin Kuo Jing Teo, Alex R. Cook

**Affiliations:** ^1^ Saw Swee Hock School of Public Health National University of Singapore and National University Health System Singapore; ^2^ Department of Statistics University of Chicago Chicago Illinois USA; ^3^ University of North Carolina Project – China Guangzhou China; ^4^ London School of Hygiene and Tropical Medicine London UK

**Keywords:** modelling, key and vulnerable populations, men who have sex with men, sex workers, transgender people, stigma

## Abstract

**Introduction:**

Singapore lacks robust data on the sizes of the key populations that are most at risk for HIV. Using the network scale‐up method for hidden or hard‐to‐reach populations, we estimate the sizes of five key populations—male clients of female sex workers (MCFSW), men who have sex with men (MSM), female sex workers (FSW), people who inject drugs (PWID) and transgender people—and profile the ages and ethnicities of respondents with the high‐risk contacts they report knowing.

**Methods:**

We conducted a cross‐sectional online survey between March and May 2019 (*n* = 2802) using a network scale‐up instrument previously developed for Singapore. Participants were recruited using an existing panel and online advertising, and the sample reweighted by age, sex, ethnicity and education attained to represent the general adult population. We built a Bayesian hierarchical model to estimate the sizes of the five key populations for HIV in Singapore.

**Results:**

After adjustment, the sizes of the at‐risk populations are estimated to be: 76,800 (95% credible interval [CI]: 64,200–91,800) MCFSW; 139,000 (95% CI: 120,000–160,000) MSM; 8030 (95% CI: 3980–16,200) FSW; 3470 (95% CI: 1540–7830) PWID and 18,000 (95% CI: 14,000–23,200) transgender people. Generally, men reported knowing more people in all the high‐risk groups; older people reported knowing more MCFSW, FSW and transgender people; and younger people reported knowing more MSM. There was a bimodal effect of age on those who reported knowing more PWIDs: people in their 20s and 60s reported more contacts.

**Conclusions:**

This study demonstrates that a size estimation study of hidden populations is quickly and efficiently scalable through using online surveys in a socially conservative society, like Singapore, where key populations are stigmatized or criminalized. The approach may be suitable in other countries where stigma is prevalent and where barriers to surveillance and data collection are numerous.

## 1 INTRODUCTION

In recent years, HIV incidence and AIDS‐related deaths have become a particular concern in hitherto lower prevalence countries in Africa, Latin America, Eastern Europe and the Asia‐Pacific, largely due to global resources to control the HIV/AIDS epidemic being funnelled into higher prevalence countries [1]. However, the low national prevalence rates in many countries mask high prevalence and incidence rates among key populations [2, 3, 4, 5, 6, 7], often stigmatized or criminalized groups, such as commercial sex workers, their clients, men who have sex with men (MSM), transgender people and people who inject drugs (PWID). These sub‐populations are particularly vulnerable to infection, and often lack access to HIV prevention and treatment services as a result of stigma and discrimination [8]. Members of key populations made up approximately 80% and about a quarter of incident HIV cases outside of and within sub‐Saharan Africa, respectively [9].

In Singapore, although the overall prevalence of HIV infections is low with fewer than 9000 cases in a population of about 5.7 million people, within sub‐populations, such as MSM, the prevalence is disproportionately high, accounting for about 40% of all cases and 57% of new diagnoses in 2019 [10]. Over 90% of all new infections reported among residents in the city‐state since 2004 have been in men [10]. Heterosexual transmissions typically occur between female sex workers (FSW) and their clients [10]. These groups, as well as transgender people and PWID, are, therefore, key populations to target prevention, diagnosis and treatment campaigns, and have been highlighted in the national action plan to eliminate HIV transmission [11]. Non‐medicinal drug use is illegal in Singapore, and while sex work, being transgender and sex between men are legal, these are subject to social stigma as in many cultures [12, 13, 14, 15, 16, 17, 18]. Sex between men was only recently legalised and was illegal at the time this study was conducted.

Despite the concentration of HIV infections within these groups, Singapore as yet does not have reliable data on the sizes of these key populations. The best estimates of the sizes of these groups come from a pilot study conducted by some of the authors [19]. However, the estimates generated from that pilot had large residual uncertainty and low precision because of the small size of the study. Without precise and accurate estimates of the sizes and demographics of the key populations, designing, implementing and evaluating the effectiveness of interventions targeted at these populations is hampered [20, 21, 22, 23]. However, stigma and criminality complicate the estimation of population sizes, as individuals may be reticent to disclose membership of these groups [22, 24].

To overcome this non‐response bias, our study uses the network scale‐up method (NSUM) to estimate the sizes of five key (or *hidden*) populations at risk for HIV in Singapore—male clients of female sex workers (MCFSW), MSM, FSW, PWID and transgender people. NSUM is an approach that has been developed for estimating the sizes of hidden or hard‐to‐reach populations and does not require participants to disclose their membership of any group, a major limitation of direct methods [25, 26]. It is, therefore, especially useful for conservative societies like Singapore and some other Asian countries where norms of saving face, piety and conformity are widespread and for which social desirability bias may be substantial. Another advantage that it can have over direct methods, such as respondent‐driven sampling, is that the same study can yield estimates of the size of multiple key populations at no additional cost, which when combined with the ease of access to members of the general population through survey platforms or direct advertising makes the NSUM method a cost‐efficient methodology, especially for resource‐constrained settings.

In spite of some limitations, such as *transmission error*, in which membership of the group is not communicated to participants, and *barrier effects*, under which some groups are preferentially selected out of the sample, this method has been shown to be effective in estimating the sizes of key populations in several other countries [27, 28, 29]. Most studies using NSUM have estimated only the size of the hidden populations, but the approach lends itself well to eliciting richer information by relating the results to the demographics of participants.

In this scale‐up study, we conducted a larger survey of around 3000 Singapore residents using an adaptation of the localized NSUM instrument piloted in a previous study [19]. The study aims to provide more precise estimates of the sizes of five key populations at risk for HIV in Singapore.

## 2 METHODS

We fielded a survey to participants drawn from an online panel and recruited through online advertising and developed a hybrid model that combines the Bayesian paradigm and bootstrap to estimate the sizes of the identified at‐risk populations for HIV in Singapore. The approach is described below.

### 2.1 Questionnaire design

We adapted an NSUM instrument developed in consultation with stakeholders involved in Singapore's HIV prevention efforts [19] and pilot tested to ensure suitability and interpretability in the Singapore context. The development of the initial questionnaire is summarized in Supplement 2 and fully detailed in the pilot study [19]. Minor changes were made for the current study, and the survey was pilot tested after the changes were made (details of the changes are provided in Supplement 2). Please see Supplement 1 for the full questionnaire used for our study.

### 2.2 Data source

Participants were recruited from the Singapore Population Health Studies (SPHS) Online Panel and via targeted Facebook advertisements. The SPHS Online Panel is an initiative supported by the National Medical Research Council to establish an online research panel that is representative of the general population of Singapore to facilitate population health research on diverse topics. The members of the online panel are anglophone Singaporeans or Permanent Residents, aged 21 years and above. We received 653 responses from the panel and supplemented these by recruitments through advertisements placed on Facebook to specified demographic segments, which we dynamically modified over the study period to obtain a sample with similar demographics to the population, to reach a desired sample size of ∼3000. Inclusion criteria for participants recruited through this route were Singapore residents aged 18 years and above. Figure S1 (Supplement 2) shows the advertisements used.

### 2.3 Data collection

Data were collected using one‐time self‐administered online surveys available in Chinese, English and Malay. Each survey took approximately 20 minutes to complete and participants were to answer all questions. Recruitment was done between March and May 2019 (2019‐03‐18 to 2019‐05‐30). Responses were screened to minimize threats to the validity of our study by removing acquiescence bias responses (participants’ tendency to answer in the affirmative to questions regardless of their true views) and its opposite, to the extent that they contradicted themselves [30, 31], or to have reported an unexpectedly large number of contacts (over 30) from one or more groups. Participants were given S$10 (∼USD7.50) as a token of appreciation for their time in the form of a direct money transfer to members of the panel or an e‐voucher to those recruited through advertising. The study received ethics approval from the NUS Institutional Review Board [NUS IRB reference: S‐19‐060]. While documented informed consent was waived by the IRB, individuals were asked to indicate their agreement to participate by clicking on the “Participate” icon offered through the online survey.

### 2.4 Sampling weights

Post‐hoc sample weights were derived to adjust the sample to better match the Singapore population aged 18 years and above. The numbers within 10‐year age bands, split by sex, ethnicity (Chinese, Indian, Malay or other) and highest level of education attained (non‐university or university) were obtained for both sample and population using Singapore's Census of Population 2020 [32]. Weights were then calculated as the ratio of population to sample counts. Additional details on the sampling process are available in Supplement 2.

### 2.5 Measures

Basic demographic information, including age, sex, citizenship status, education and religion, was collected. Participants were asked to rate the acceptability of 13 different behaviours based on their own opinions on a 10‐point Likert scale. For the NSUM component of the questionnaire, participants were to provide estimates of the number of people they knew in 10 known populations used as reference groups (tabulated in Table S1 in Supplement 2) and in the five hidden populations. Respondents who indicated that they knew people in the at‐risk groups were also asked about the age profiles and ethnicities of these contacts. We used the recommended 10 known populations out of the 20 that were used as reference groups in the pilot study based on leave one out validation [33]. An additional key population (transgender people) was added to the four key populations (MCFSW, MSM, FSW and PWID) used in the pilot study. For an individual to be considered to be a participant's contact, the person must be known to the participant by name and sight, and vice‐versa; the contact must be currently residing in Singapore; and lastly, the participant should have spoken to their contact in person or via a mobile device (text messages or phone call) at least once in the last 1 year.

### 2.6 Demographics of participants knowing high‐risk groups

We estimated the relationship between participants’ age, sex and ethnic group on the number of people known in each of the 10 known and five hidden groups using generalized additive models, taking advantage of their flexibility to accommodate possibly non‐linear relationships. A spline on age was used with stratification by gender or ethnicity (excluding any non‐Chinese, non‐Indian or non‐Malay participants, who were few in number). This provides an indirect estimate of the make‐up of each of the high‐risk groups.

### 2.7 Statistical analyses

We modified the Bayesian NSUM approach previously developed by Teo et al. [19] to accommodate sample weighting and thereby partially overcome barrier effects caused by groups in the population being less likely to be included in the sampling frame. The number of *i*’s contacts in reference population *j*, $N_{ij}^{R}$, was modelled to have a Poisson distribution with mean $\lambda \alpha_{i}S_{j}^{R}$, where $S_{j}^{R}$ is the (known) reference population size; $\alpha_{i}∼\mathrm{logN}\left(0,\theta \right)$ a random effect with θ∼U(0,10); and λ a scaling parameter. The model for hidden population *j* is similar, $N_{ij}^{H}\;∼\;Po\left(\lambda \alpha_{i}\tau_{ij}S_{j}^{H}\right)$, but in the *adjusted* model has an extra term for transmission error $\tau_{ij}$ and with $S_{j}^{H}$ unknown. The *unadjusted* model sets $\tau_{ij}$ to 1 to remove this effect. The extra term in the adjusted model accounts for variability in the number of contacts belonging to the hidden populations whose membership is known by the participant, which we model as a function of participants’ social acceptability score for the population, $x_{ij}$. As in the previous study, we assume $\tau_{ij}=\exp\left\{\upsilon_{j}\left(x_{ij}-U_{j}\right)\right\},\;$ where $U_{j}$ is the highest acceptability score the questionnaire permits. The implicit assumption, therefore, is that those with maximal social acceptability for population *j* know the membership of all their contacts belonging to this group, while those with lower acceptability know a fraction, estimated from the data.

Parameters were estimated under a Bayesian approach [34]. Non‐informative priors were assigned to hidden population sizes $S_{l}^{H}$, the scaling parameter λ, transmission error adjustment parameters $\upsilon_{j}$ and random effects hyperparameter θ (as tabulated in Table S2 in Supplement 2). Posteriors were sampled using Just Another Gibbs Sampler [35] with 50,000 iterations and the first 10% discarded as burn‐in. Convergence was assessed based on Geweke's [36] and Heidelberger and Welch's diagnostics [37]. We also performed a sensitivity analysis using the estimates from the pilot study as informative priors which yielded very similar results (see Table S3 and Figures S7 and S9 in Supplement 2).

To derive estimates from a sample that better matched the Singapore population, we first bootstrapped the dataset 100 times, resampling with replacement proportional to the sampling weights. We then fit the Bayesian model described above to each of the resampled data and used Rubin's method [38] to combine the population size estimates from the 100 resampled datasets before converting back to the natural scale, as described in Supplement 2.

Estimation of the total personal network size was done in the same way, through the product of λ and the total population living in Singapore (5.7M).

## 3 RESULTS

After data cleaning, responses of 2802 participants remained from 3225 initial responses (653 from the online panel; 2572 through online advertising). Table 1 shows participants’ demographics. The sample after reweighting is mostly representative of the general population, although it contains slightly too few Buddhists and too many Christians than the population in the 2020 census expects.

**Table 1:** Demographics of study participants pre‐ and post‐reweighting

Demographic variables	Pre *N* (%)	Post *N* (%)	Singapore population (2020 census)
	*N =* 2802	*N =* 2802	
Age, in years			
Mean (SD^a^)	34.7 (13.2)	46.3 (13.8)	47.5
Median (range)	30 (18–92)	49 (18–92)	
Sex			
Female	1639 (58.5)	1464 (52.3)	51.5%
Male	1163 (41.5)	1338 (47.7)	48.5%
Citizenship status			
Singaporean	2547 (90.9)	2537 (90.5)	86.3%
Permanent Resident	205 (7.3)	221 (7.9)	13.7%
Ethnicity			
Chinese	2441 (87.1)	2127 (75.9)	75.8%
Indian	180 (6.4)	236 (8.4)	8.5%
Malay	142 (5.1)	356 (12.7)	12.6%
Marital status^d^			
Currently married	972 (34.7)	1670 (59.6)	62.8%
Never married	1693 (60.4)	861 (30.7)	26.9%
Divorced/Separated	99 (3.5)	195 (6.9)	4.6%
Widowed	19 (0.7)	50 (1.8)	5.7%
Religion^d^			
Buddhism	709 (25.3)	632 (22.6)	31.5%
Christianity	787 (28.1)	831 (29.7)	18.9%
No religion	725 (25.9)	525 (18.7)	19.8%
Islam	170 (6.1)	381 (13.6)	15.2%
Taoism	196 (7.0)	206 (7.3)	9.1%
Hinduism	89 (3.2)	115 (4.1)	4.9%
Others	126 (4.5)	113 (4.0)	0.6%
Education attained^b^, ^e^			
Non‐university	1315 (46.9)	1683 (60.1)	61.0%
University	1487 (53.1)	1119 (39.9)	39.0%
Household income^f^			
Less than $2000	245 (8.7)	307 (11.0)	8.2%
$2000–$3999	478 (17.1)	503 (18.0)	12.1%
$4000–$5999	478 (17.1)	503 (18.0)	12.1%
$6000–$10,000	694 (24.8)	738 (26.3)	21.6%
More than $10,000	529 (18.9)	462 (16.5)	46.0%
Housing type^c^, ^g^			
1/2‐room HDB	57 (2.1)	80 (2.9)	6.5%
3‐room HDB	327 (11.7)	378 (13.5)	17.7%
4‐room HDB	897 (32.0)	912 (32.6)	31.6%
5‐room HDB	877 (31.3)	946 (33.8)	22.9%
Condominium	401 (14.3)	316 (11.3)	16.0%
Landed property	176 (6.3)	115 (4.1)	5.0%

^a^ SD is the sample standard deviation.

^b^ Education attained represents an individual's highest level of education attained. Non‐university represents all levels of education, including primary, secondary, pre‐university, vocational schools and polytechnics.

^c^ Housing type was used as a proxy for socio‐economic status. The majority of the Singaporean population live in HDB (Housing Development Board) flats which are Singapore's primary public housing units. Private housing includes condominiums and landed properties (which are standalone houses). The wealthier population tend to live in either large HDB flats (4–5‐room HDB flats), condominiums or landed properties, hence, an individual's housing type is a good proxy for their socio‐economic status in Singapore. All statistics reported for the Singapore population represent those for the population aged 18 and older unless indicated otherwise.

^d^ Data for Singapore population are for those aged 20 and above.

^e^ Education data are for the resident population aged 15 years and older.

^f^ Monthly household income data are for households with at least one employed person in the household, no age breakdown.

^g^ Housing type is for the resident population, no age breakdown.

**Figure 1:**
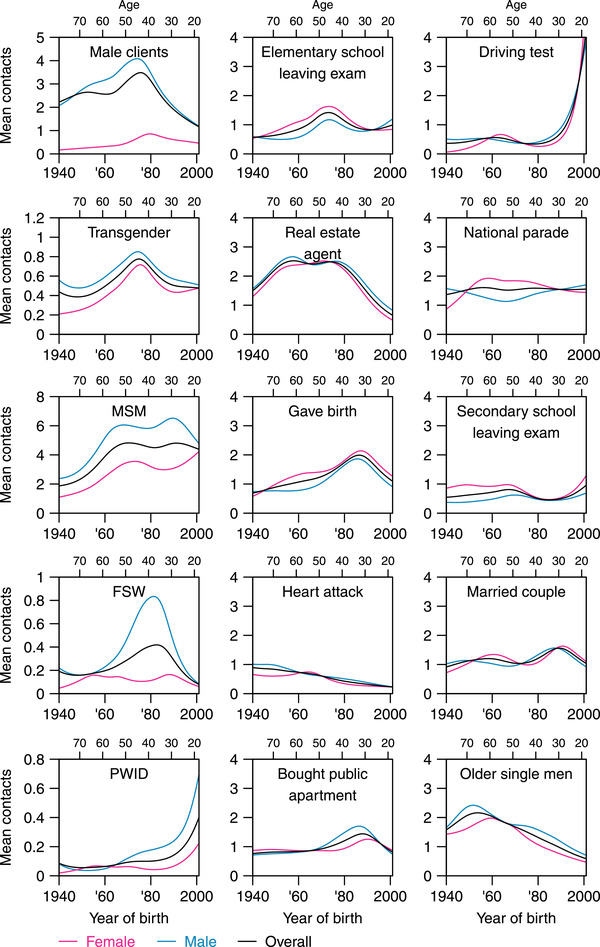
Mean number of contacts and participants’ year of birth grouped by sex for the five key populations and 10 reference populations. The plots in the first column represent the five key populations and those in the second and third columns represent the 10 reference groups. Note the difference in the scales for the mean number of contacts for the key populations and for the reference groups. The black line in each plot represents the mean number of contacts for the total sample, the blue line represents the mean number of contacts for male respondents and the pink line represents the mean number of contacts for female respondents. *Male clients, male clients of female sex workers; Transgender, transgender individuals; MSM, men who have sex with men; FSW, female sex workers; PWID, people who inject drugs; Elementary school leaving exam, represents students who sat for the elementary school (the equivalent of primary school) leaving examinations in 2018 taken around age 12 in Singapore; Bought public apartment, those who bought a public apartment in Singapore in 2017; National parade, those who attended Singapore's annual ticketed Independence Day parade in 2018; Secondary school leaving exam, represents students around age 16 who sat for these examinations in 2018 at the end of their secondary school education (the equivalent of junior high school); Married couple, represents couples who got married in 2018*.

**Figure 2:**
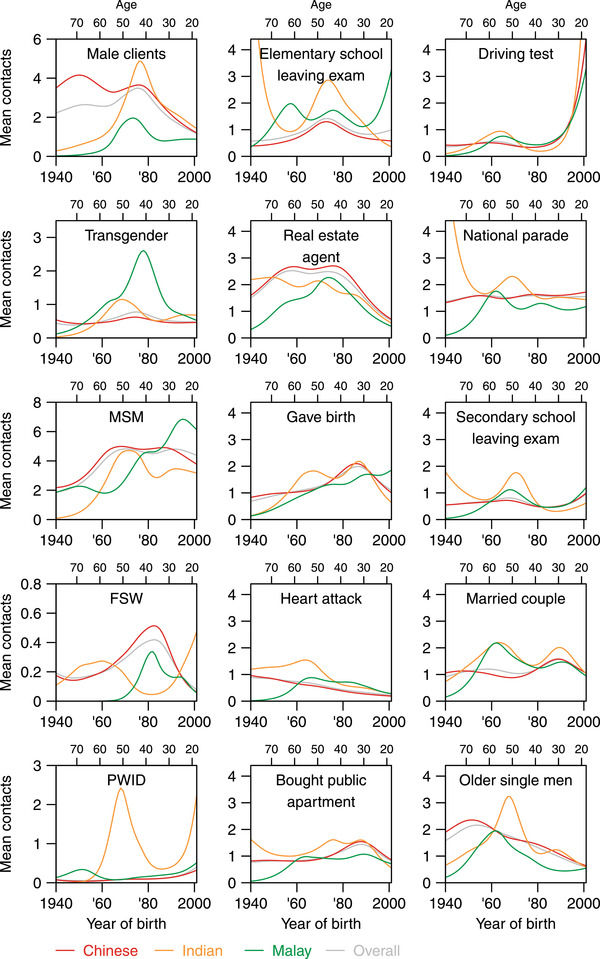
Mean number of contacts and participants’ year or birth grouped by ethnicity for the five key populations and 10 reference populations. The plots in the first column represent the five key populations and those in the second and third columns represent the 10 reference groups. Note the difference in the scales for the mean number of contacts for the key populations and for the reference groups. The grey line in each plot represents the mean number of contacts for the total sample, the red line represents the mean number of contacts for Chinese respondents, the green line represents Malay respondents and the saffron line represents Indian respondents. *Male clients, male clients of female sex workers; Transgender, transgender individuals; MSM, men who have sex with men; FSW, female sex workers; PWID, people who inject drugs; Elementary school leaving exam, represents students who sat for the elementary school (the equivalent of primary school) leaving examinations in 2018 taken around age 12 in Singapore; Bought public apartment, those who bought a public apartment in Singapore in 2017; National parade, those who attended Singapore's annual ticketed Independence Day parade in 2018; Secondary school leaving exam, represents students around age 16 who sat for these examinations in 2018 at the end of their secondary school education (the equivalent of junior high school); Married couple, represents couples who got married in 2018*.

Table 2 shows the adjusted and unadjusted size estimates of the five key populations from the Bayesian modelling. We estimated an individual's total personal network size to be 142 (95% credible interval [CI] 134–150). The adjusted sizes of the at‐risk populations are estimated to be: 76,800 (95% CI: 64,200–91,800) MCFSW; 139,000 (95% CI: 120,000–160,000) MSM; 8030 (95% CI: 3980–16,200) FSW; 3470 (95% CI: 1540–7830) PWID and 18,000 (95% CI: 14,000–23,200) transgender people. On average, participants had more contacts who were MSM or MCFSW than other high‐risk groups (Figures 1 and 2).

**Table 2:** Adjusted and unadjusted size estimates of the five key populations from the Bayesian modelling

Population	Unadjusted size^b^	95% CI^a^	Adjusted size^c^	95% CI^a^
MCFSW	29,900	(27,000–33,100)	76,800	(64,200–91,800)
Transgender	9190	(7980–10,600)	18,000	(14,000–23,200)
MSM	44,000	(40,200–48,300)	139,000	(120,000–160,000)
FSW	5130	(3790–6940)	8030	(3980–16,200)
PWID	1810	(1410–2320)	3470	(1540–7,830)
Personal network	142	(134–150)	142	(134–150)

Note: Adjusted estimates use social‐acceptance scores to adjust estimates, as described in the Methods section; unadjusted estimates do not.

Abbreviations: FSW, female sex workers; MCFSW, male clients of female sex workers; MSM, men who have sex with men; PWID, people who inject drugs.

^a^ 95% CI represents the credible interval of the mean estimates.

^b^ Unadjusted size estimates were derived accounting for barrier effects but not transmission error.

^c^ The adjusted size estimates were derived after accounting for transmission error and barrier effects.

There were strong age and sex effects observed for respondents and their contacts in the high‐risk groups. Male respondents knew more people in the high‐risk groups on average; older people knew more MCFSW, FSW and transgender people; and younger people knew more MSM (Figure 1). Specifically, men in their 40s to 60s knew more FSW and more of their clients, and younger men in the late 20s and early 30s knew more MSM. Older men in their 50s and 60s also knew more transgender people (Figure 1). There was a bimodal effect of age on knowing PWID, with both younger males in their early 20s and older males in their 50s and 60s reporting higher numbers of contacts with PWID (Figure 1).

We also observed some differences between the main ethnic groups in Singapore in the number of contacts they reported knowing in the high‐risk groups. Generally, Malays reported knowing more MSM with younger Malays reporting the greatest number of MSM contacts on average (Figure 2). More Indians and Malays reported higher numbers of transgender and PWID contacts, and more Indians generally reported more FSW contacts (Figure 2).

Participants’ ratings on the social acceptability of the 13 behaviours are illustrated in Figures S2–S5 and summarized in Tables S4–S7 (Supplement 2). Overall, respondents found injecting illicit drugs to be highly unacceptable along with drink‐driving, racist behaviours and spitting in public (Figure S2). Of all the high‐risk groups, participants were more accepting of transgender people. The majority of respondents reported finding homosexuality unacceptable, rating it as the second least acceptable out of the other key populations (Figure S2). Between sexes, males are more accepting of commercial sex work than are females, rating both FSW and MCFSW more favourably (Figure S3). Although both sexes rated injecting drugs as very unacceptable, females found it more unacceptable (Figure S3). Compared to older individuals, we found that younger people were generally more accepting of the behaviours of members in the five key populations, especially for homosexual behaviour and transgender people (Figure S4). They also rated sex work as slightly more acceptable than older people did on average (Figure S4).

## 4 DISCUSSION

This study provides the first large‐scale network scale‐up survey of high‐risk populations for HIV in Singapore and one of the first in Southeast Asia [39, 40]. Unique to our study were relating the demographics of respondents to their contacts in the key populations, which was possible due to the large sample size. Our study provides estimates for the transgender population, heretofore uncounted, and our estimates were consistent with the estimates from the pilot study [19], but with greater precision.

Despite the overlapping CIs, there were, nevertheless, notable differences in the point estimates for MSM, PWID and FSW between our study and the pilot. In this study, we estimated there to be 139,000 (95% CI: 120,000–160,000) MSM compared to 210,000 (95% CI: 140,000–300,000) MSM from the pilot study [19]. The UNAIDS median prevalence estimate of MSM for low‐ and middle‐income countries in Asia and the Pacific is 1.63% [IQR: 0.26–3.10%] of the male population [41] and a scale‐up study in Japan, a high‐income country comparable to Singapore, estimated a prevalence of 2.9% MSM among the total male population [42]. Our current estimates put the proportion of MSM at about 2.5% of the total resident population of ∼5.7 million (or ∼5% of the male population) compared to about 3.7% (∼7% of the male population) for the pilot. A possible explanation for differences in the two studies is a non‐linearity between age and the number of people known in the MSM group, which was previously assumed.

For PWID, this study estimates there to be 3470 (95% CI: 1540–7830) PWID compared to 11,000 (95% CI: 6500–17,000) PWID from the pilot study [19]. In both cases, despite efforts to make the sample representative (through posthoc reweighting in the current study), the size estimates for this sub‐population may be biased downwards as incarcerated PWID may not meet the definitions of a contact used to elicit the social network of our participants. In Singapore, this puts the estimated prevalence of PWID at 0.03 per 100,000 population compared to 110 per 100,000 population in Thailand [39], which may reflect the strictness of Singapore's drug enforcement.

The average number of contacts in the reference groups known by our respondents—broken down by age and sex—is in strong accordance with *a priori* expectations. These findings lend a degree of face validity to the results on the demographic structure of the key populations. We found both age and sex effects in the number of contacts belonging to these groups. Older males in their 40s to 60s know more MCFSW, and FSW themselves, which comports with previous work showing clients of commercial sex workers in Singapore tend to be older men aged 30 and above [43, 44]. Older males in their 50s and 60s also knew more transgender people, possibly attributable to some transgender women being involved in sex work. In contrast, younger Singaporeans knew more MSM and typically viewed MSM as being more socially acceptable, which may reflect changing societal norms and differing rates of sexual orientation disclosure across age groups [45, 46, 47]. Differences between the number of contacts in the hidden groups were also present between ethnic groups and may reflect structural inequities or social factors that disproportionately impact various groups. For instance, Malay respondents knew more drug users—which parallels the higher levels of incarcerations for drug offences in Singapore's Malay community [48, 49]—but fewer sex workers. Malays and Indians also knew more transgender people, potentially signalling a disproportionate number of transgender people of these ethnicities.

One limitation of our study is caused by the high proportion of non‐resident foreigners in Singapore with different legal statuses from locals and resident foreigners: as it is not always clear which residency status contacts have, we did not ask this in the network scale‐up instrument, and thus cannot estimate the share of the high‐risk groups who are local, resident foreigners or non‐resident. In addition, our recruitment method may have biased respondents towards those with higher technological literacy, and although reweighting controlled for any initial imbalance in age, it does not control for bias within age groups against those with lower technological capital. We accounted for transmission error by implicitly inflating the number of contacts among those with less accepting views of the hidden populations, as in our previous study [19]. While this comported with the observed differences in reported numbers of contacts, the form of this inflation may not have been perfectly represented, and more research on this would be valuable. It is also possible that some groups may be subject to transmission error beyond what we are able to incorporate, such as older individuals being less likely to disclose being MSM. The groups for which the adjustment had the greatest effect were MCFSW, transgender and MSM, and so the estimates of these groups may be more prone to mis‐specification. Future studies may want to consider other study design‐based approaches involving sampling from the hidden populations as well if feasible (although this is a deviation from the design of the basic scale‐up model itself) and using a generalized scale‐up estimator to overcome the limitations of the basic scale‐up estimator [50].

## 5 CONCLUSIONS

Despite these limitations, we have demonstrated through our study that a size estimation study of hidden populations is efficiently scalable through using online surveys. By fielding the survey anonymously and online, it was possible to quantify stigma and adjust estimates accordingly, which is an important feature for socially conservative populations. As many countries in Southeast Asia did not meet the 2020 UNAIDS 90‐90‐90 target due to issues of stigma and insufficient resources among others [3], our study highlights a cost‐efficient approach which circumvents the challenges of stigma to estimate the sizes of the populations at risk for effective programming and resource allocation, as the region has high internet and Facebook penetration rates [51].

The approach may, therefore, also be useful in other Asian settings, including low‐ and middle‐income countries, where internet use is widespread, to improve their resource allocation and aid their control of the HIV/AIDS epidemic.

## COMPETING INTERESTS

There are no competing interests to declare.

## AUTHORS’ CONTRIBUTIONS

ARC conceptualized the study. ARC, AKJT, KP and SEDQ contributed to the study design. SEDQ did the data collection. ARC, YC, JRK and SEDQ contributed to the statistical analyses and making of the figures. SEDQ and RKJT conducted the literature review. SEDQ, YC and ARC wrote the initial draft. All authors contributed equally to interpreting the data, critically reviewing the manuscript and approving the final version.

## FUNDING

This study was funded by the Singapore Population Improvement Centre (SPHERiC) (NMRC/CG/C026/2017_NUHS) and supported by the Singapore Population Health Studies.

## Supporting information


**Supplement 1**: This file contains the questionnaire that was used for data collection of the study.

**Supplement 2**: This file contains additional details on the study's methodology and some additional tables of parameters and figures of results that are not the main outcomes but may offer some more insight into the study's findings if required.

## Data Availability

Research data are not shared as participants did not consent to having their data made publicly available.
