# Supplementary material for: Application of the network scale‐up method to estimate the sizes of key populations for HIV in Singapore using online surveys
Source: J Int AIDS Soc. 2023 Mar 15;26(3):e25973. doi: 10.1002/jia2.25973 (PMC10015632; doi:10.1002/jia2.25973)
Supplement: Supplementary file 1 — Supplement 1: This file contains the questionnaire that was used for data collection of the study. [file JIA2-26-e25973-s002.docx]

**Survey questions for the study titled “Estimating the sizes of key populations for HIV in Singapore using the network scale-up method with data from online surveys”. Notes are added in yellow for the benefit of international readers: these were not provided in the questionnaire taken by participants.**

**Section 1: Basic Demographics**

**1. In which year were you born? _____________**

**2. What is your sex?**

| a. | Male |
| --- | --- |
| b. | Female |

**3. What is your ethnicity?**

| a. | Chinese |
| --- | --- |
| b. | Malay |
| c. | Indian |
| d. | Others (please specify): _____________ |

**4. What is your marital status?**

| a. | Never married |
| --- | --- |
| b. | Currently married |
| c. | Separated but not divorced |
| d. | Divorced |
| e. | Widowed |
| f. | Refuse to answer |

**5. Do you have any children?**


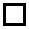
 1. Yes


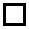
 2. No (skip to Q8)

888. Refuse to answer (skip to Q8)


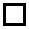


**6. Which age groups are your children in? You may select more than one option.**

| 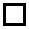 1. Under the age of 5  How many children under the age of 5 do you have? |
| --- |
| 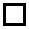 2. Between the age of 5 and 12  How many children between the age of 5 and 12 do you have? |
| 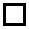 3. Between the age of 13 and 30  How many children between the age of 13 and 30 do you have? |
| 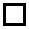 4. 31 years and older  How many children 31 years and older do you have? |

**7. Do you have any grandchildren?**


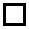
 1. Yes


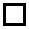
 2. No

888. Refuse to answer


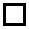


**8. What is the highest level of education that you have attained?**

| a. | No formal qualifications/lower primary |
| --- | --- |
| b. | Primary (PSLE) |
| c. | Secondary (‘O’/’N’ Level) |
| d. | ITE/NTC |
| e. | ‘A’ Level/Polytechnic/Diploma |
| f. | University |
| g. | Others (please specify): ___________________ |
| h. | Refuse to answer |

**Notes: the PSLE is the primary school leaving exam that Singaporean children take aged around 12 to progress to secondary school. The O or N levels are exams taken around age 16. ITE/NTC are vocational schools focusing on technical skills. A levels and polytechnic qualifications are taken at advanced secondary level around age 18 or 19.**

**9. What is your total household income per month on average?**

***Note: Household includes persons living in the same house, excluding tenants, housemates and domestic helpers. Income includes salaries, bonuses, rental income and other forms of monetary income.***


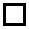
 1. Less than $2000


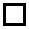
 2. $2000 to $3999


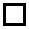
 3. $4000 to $5999


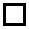
 4. $6000 to $10 000


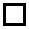
 5. More than $10 000


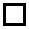
 888. Refuse to answer


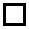
 999. Do not know

**Note: 1 SGD** $\approx$ **0.75 USD.**

**10. What type of housing do you live in?**

| a. | 1-room HDB |
| --- | --- |
| b. | 2-room HDB |
| c. | 3-room HDB |
| d. | 4-room HDB |
| e. | 5-room HDB/executive flat |
| f. | Condominium |
| g. | Executive condominium |
| h. | Landed-property |
| i. | Others (please specify): _______________ |
| j. | Refuse to answer |
| k. | Do not know |

**Note: Most Singaporean households live in public housing built by the housing development board (HDB). Larger numbers of rooms is used as a proxy for socio-economic status.**

**11. What is your religion? (you can select more than one)**

| a. | Buddhism |
| --- | --- |
| b. | Taoism/Chinese Traditional Beliefs |
| c. | Christianity (including Roman Catholics, Protestants, etc.) |
| d. | Islam |
| e. | Hinduism |
| f. | Sikhism |
| g. | No religion |
| h. | Others (please specify): ________________ |

**12. I am a…**

| a. | Singaporean |
| --- | --- |
| b. | Permanent Resident |
| c. | Others (please specify): _____________ |

**Section 2: Social Acceptability**

In this section, please rate the acceptability of certain behaviours according to your own views/opinions on a scale of **1 – 10, with 1 = "Totally Unacceptable" and 10 = "Totally Acceptable"**. For each question below, please select the number that best corresponds with your opinion.

|  | Totally Totally unacceptable acceptable | | | | | | | | | |
| --- | --- | --- | --- | --- | --- | --- | --- | --- | --- | --- |
| 1. A woman who breastfeeds publicly | 1 | 2 | 3 | 4 | 5 | 6 | 7 | 8 | 9 | 10 |
| 2. Someone who makes racist comments | 1 | 2 | 3 | 4 | 5 | 6 | 7 | 8 | 9 | 10 |
| 3. A man who has sex with another man | 1 | 2 | 3 | 4 | 5 | 6 | 7 | 8 | 9 | 10 |
| 4. A woman who has sex with another woman | 1 | 2 | 3 | 4 | 5 | 6 | 7 | 8 | 9 | 10 |
| 5. Someone driving while drunk | 1 | 2 | 3 | 4 | 5 | 6 | 7 | 8 | 9 | 10 |
| 6. A man who pays for sex with a woman | 1 | 2 | 3 | 4 | 5 | 6 | 7 | 8 | 9 | 10 |
| 7. A person who had a sex change | 1 | 2 | 3 | 4 | 5 | 6 | 7 | 8 | 9 | 10 |
| 8. A woman who smokes cigarettes | 1 | 2 | 3 | 4 | 5 | 6 | 7 | 8 | 9 | 10 |
| 9. Someone who spits in public | 1 | 2 | 3 | 4 | 5 | 6 | 7 | 8 | 9 | 10 |
| 10. A woman who sells sex | 1 | 2 | 3 | 4 | 5 | 6 | 7 | 8 | 9 | 10 |
| 11. A woman who is pregnant before marriage | 1 | 2 | 3 | 4 | 5 | 6 | 7 | 8 | 9 | 10 |
| 12. Someone who takes non-medical drugs by injections | 1 | 2 | 3 | 4 | 5 | 6 | 7 | 8 | 9 | 10 |
| 13. Someone who puts their parents in a nursing home | 1 | 2 | 3 | 4 | 5 | 6 | 7 | 8 | 9 | 10 |

14. Have you heard of Penal Code Section 377A before?

a. Yes

b. No

15. **Penal Code *section 377A* is a legislation in Singapore that criminalizes sex between two men performed both publicly and privately. However, except in rare cases, it is not enforced.**

**In your opinion, which level of enforcement of *section* 377A would you prefer? Please rank the following statuses in order of your preference, with ‘1’ being your most preferred option and ‘3’ being your least preferred option.**

a. Maintained as is (i.e. it is illegal for a man to have sex with another man, but no action will be taken against them as long as they do it in private.)

b. Removed (i.e. men who have sex with other men will be treated in the same way as heterosexual couples and women who have sex with other women.)

c. Enforced (i.e. it will remain illegal for a man to have sex with another man, and the police will actively enforce this law.)

**Section 3: Size of Social Networks**

**To plan effective HIV prevention and treatment responses, we first need to estimate the number of people who may be at risk of HIV in Singapore. Therefore, please answer the following questions as accurately as possible.**

**The people you know from each group/category should meet all three of the following criteria:**

1. **People you know by sight and name, and who also know you by sight and name;**
2. **People living in Singapore; and**
3. **People you have spoken to in person or via a mobile device (text messages or phone call) at least once in the last one year.**

***Example: How many medical doctors (excluding dentists) do you know?***

***Assuming you know 3 doctors in total:***

***Dr Hanif: your neighbour, whom you spoke to this morning***

***Dr Alice: your friend who lives in Malaysia now, but whom you text weekly***

***Dr Chen: your GP whom you saw last week for a check-up***

***In this scenario, you would enter “2” instead of “3” since Dr Alice does not meet all the above criteria.***

**Note: If you do not know anyone belonging to a particular group/category, please enter “0” as your answer.**

|  | **Total number** |
| --- | --- |
| 1. How many students do you know who took the PSLE in 2018? |  |
| 1. How many licensed property agents do you know? |  |
| 1. How many men do you know who have ever paid for sex in Singapore?   Here participants would answer branching questions about the age group and ethnicity of the people they know in this category if their answer is not **‘0’**. For example, if a participant indicates that he/she knows **‘2’** men who have ever paid for sex in Singapore, this is what he/she would see:  *“You have reported that you know 2 men who have ever paid for sex in Singapore. Think about these 2 people. Please tell us a bit more about them:*   1. *What is the age range of person 1? If you are unsure, select the age range of person 1 to your best knowledge. 19 years old or less / 20-29 years old / 30-39 years old / 40-49 years old / 50-59 years old / 60-69 years old / 70 years old and above* 2. *What is the ethnicity of person 1? Chinese / Malay / Indian / Others (please specify:)* 3. *What is the age range of person 2? If you are unsure, select the age range of person 1 to your best knowledge. 19 years old or less / 20-29 years old / 30-39 years old / 40-49 years old / 50-59 years old / 60-69 years old / 70 years old and above* 4. *What is the ethnicity of person 2? Chinese / Malay / Indian / Others (please specify:)”*   This will go on up to 5 sets. If a participant’s response is more than 5, he/she will only need to fill in the details of the 5 he/she is closest to/knows best. For example, if a participant indicates that he/she knows **‘7’** men who have ever paid for sex in Singapore, this is what he/she would see:  *“You have reported that you know more than 5 men who have ever paid for sex in Singapore. Think about the 5 that you are closest to/know best. Please tell us a bit more about them:*   1. *What is the age range of person 1? If you are unsure, select the age range of person 1 to your best knowledge. 19 years old or less / 20-29 years old / 30-39 years old / 40-49 years old / 50-59 years old / 60-69 years old / 70 years old and above* 2. *What is the ethnicity of person 1? Chinese / Malay / Indian / Others (please specify:)* 3. *What is the age range of person 2? If you are unsure, select the age range of person 1 to your best knowledge. 19 years old or less / 20-29 years old / 30-39 years old / 40-49 years old / 50-59 years old / 60-69 years old / 70 years old and above* 4. *What is the ethnicity of person 2? Chinese / Malay / Indian / Others (please specify:)* 5. *What is the age range of person 3? If you are unsure, select the age range of person 1 to your best knowledge. 19 years old or less / 20-29 years old / 30-39 years old / 40-49 years old / 50-59 years old / 60-69 years old / 70 years old and above* 6. *What is the ethnicity of person 3? Chinese / Malay / Indian / Others (please specify:)* 7. *What is the age range of person 4? If you are unsure, select the age range of person 1 to your best knowledge. 19 years old or less / 20-29 years old / 30-39 years old / 40-49 years old / 50-59 years old / 60-69 years old / 70 years old and above* 8. *What is the ethnicity of person 4? Chinese / Malay / Indian / Others (please specify:)* 9. *What is the age range of person 5? If you are unsure, select the age range of person 1 to your best knowledge. 19 years old or less / 20-29 years old / 30-39 years old / 40-49 years old / 50-59 years old / 60-69 years old / 70 years old and above* 10. *What is the ethnicity of person 5? Chinese / Malay / Indian / Others (please specify:)”* |  |
| 1. How many women do you know who had a baby in 2018? |  |
| 1. How many people do you know who had a heart attack in 2018? |  |
| 1. How many people do you know who identify as transgender?   Similar branching questions here as in Q.3. |  |
| 1. How many people do you know who bought an HDB in 2018? |  |
| 1. How many people do you know who obtained a driving license in 2018? |  |
| 1. How many people do you know who attended the Singapore National Day Parade in 2018? |  |
| 1. How many men do you know whom you think are gay/homosexual?   Similar branching questions here as in Q.3. |  |
| 1. How many students do you know who took O-Levels in 2018? |  |
| 1. How many couples do you know who got married in Singapore in 2018? (Count each couple *once*) |  |
| 1. How many female sex workers do you know?   Similar branching questions here as in Q.3. |  |
| 1. How many unmarried (single and never married before) men above the age of 50 do you know? |  |
| 1. How many people do you know who inject drugs for non-medical/recreational purposes?   Similar branching questions here as in Q.3. |  |

**Section 4: Attitudes toward HIV**

Based on what you feel, please indicate your level of agreement for the following statements on a scale of **1 – 5, where 1 = "Strongly Disagree" and 5 = "Strongly Agree"** based on your own feelings.

|  | Strongly disagree Strongly agree | | | | |
| --- | --- | --- | --- | --- | --- |
| 1. I would not want to come into physical contact with people living with HIV | 1 | 2 | 3 | 4 | 5 |
| 1. I would feel uncomfortable knowing that my neighbour or a colleague (at work/school) has HIV | 1 | 2 | 3 | 4 | 5 |
| 1. I would feel uncomfortable caring for my family member (such as my child or sibling) if he/she contracts HIV | 1 | 2 | 3 | 4 | 5 |
| 1. I would not share eating utensils or a drinking glass with a person who has HIV | 1 | 2 | 3 | 4 | 5 |
| 1. I would be comfortable being tested for HIV just like being tested for my blood glucose level. | 1 | 2 | 3 | 4 | 5 |
| 1. I think if people act responsibly, they will not contract HIV | 1 | 2 | 3 | 4 | 5 |
| 1. I would not drink from a water cooler if a person with HIV had just drunk from it | 1 | 2 | 3 | 4 | 5 |
| 1. I tend to think that people living with HIV do not share the same values as me | 1 | 2 | 3 | 4 | 5 |
| 1. I fear I could contract HIV if I came into contact with the saliva of a person who has HIV | 1 | 2 | 3 | 4 | 5 |
| 1. I would be willing to care for my family member (such as my child or sibling) if he/she contracts HIV | 1 | 2 | 3 | 4 | 5 |
| 1. I think people who have HIV have usually engaged in immoral acts | 1 | 2 | 3 | 4 | 5 |
| 1. A teacher who has HIV should not be allowed to teach children | 1 | 2 | 3 | 4 | 5 |
| 1. I think Singaporeans in general tend to stigmatise people who have HIV | 1 | 2 | 3 | 4 | 5 |

14. Overall, what would you say are your general attitudes towards people living with HIV?

1. I have largely negative views on people living with HIV
2. I have largely positive views on people living with HIV
3. I have neither negative, nor positive views on people living with HIV

15. Could you please list some reasons why you have that opinion?

|  |
| --- |

**Section 5: Knowledge and Awareness of HIV**

In this section, we would like to gauge how much people know about HIV prevention, transmission and treatment. Note: If you really do not know whether the statement is true or false, please select “Do not know”.

|  |  | **True** | **False** | **Do not know** |
| --- | --- | --- | --- | --- |
|  |  |  |  |  |
| 1. | A person can get HIV …  … by being coughed or sneezed on by someone who has HIV. | T | F | DK |
| 2. | A person can get HIV…  … by sharing a glass of water with someone who has HIV. | T | F | DK |
| 3. | A person can get HIV…  … by shaking hands with someone who has HIV. | T | F | DK |
| 4. | A person can get HIV…  … through mosquito bites. | T | F | DK |
| 5. | A person can get HIV…  … by sharing a toilet with someone who has HIV. | T | F | DK |
| 6. | A person can get HIV…  … by being in a swimming pool with someone who has HIV. | T | F | DK |
| 7. | A person can get HIV…  … through getting tattoos and piercings. | T | F | DK |
| 8. | A person can get HIV…  … by sharing syringes or needles with others when injecting drugs. | T | F | DK |
| 9. | A person can get HIV…  … by deep kissing (putting their tongue in their partner’s mouth) if their partner has HIV. | T | F | DK |
| 10. | A person can get HIV…  … through oral sex (mouth on the penis/vagina). | T | F | DK |
| 11. | A person can get HIV…  … through vaginal sex (penis inside the vagina). | T | F | DK |
| 12. | A person can get HIV…  … through anal sex (penis inside the anus). | T | F | DK |
| 13. | A woman can get HIV if she has sex during her period. | T | F | DK |
| 14. | Pulling out the penis before a man ejaculates (releases sperm) keeps a woman from getting HIV during sex. | T | F | DK |
| 15. | Showering, or washing one’s genitals/private parts (penis/vagina) after sex keeps a person from getting HIV. | T | F | DK |
| 16. | Having sex with more than one partner can increase a person’s chance of getting HIV. | T | F | DK |
| 17. | A woman who has HIV can pass it on to her new-born child through breastfeeding. | T | F | DK |
| 18. | Taking an HIV test one week after having sex will tell a person if he/she has HIV. | T | F | DK |
| 19. | People who have been infected with HIV quickly show serious signs of being infected. | T | F | DK |
| 20. | People can lower their chances of getting HIV by using a condom correctly every time they have sex. | T | F | DK |
| 21. | Using Vaseline or baby oil with condoms lowers a person’s chance of getting HIV. | T | F | DK |
| 22. | Medicines are available to prevent HIV transmission from a mother to her unborn/new-born child. | T | F | DK |
| 23. | A person being treated effectively for HIV cannot spread the infection to others. | T | F | DK |
| 24. | There is a vaccine that people can get to protect them from getting HIV. | T | F | DK |
| 25. | There is a cure for AIDS. | T | F | DK |

26. Have you heard of HIV PrEP (or pre-exposure prophylaxis)?

a. Yes

b. No

27. Have you heard of HIV PEP (or post-exposure prophylaxis)?

a. Yes

b. No

**Section 6: Attitudes toward Key Populations Affected by HIV**

Based on what you feel, please indicate your level of agreement for the following statements about your attitude towards gay men and lesbian women, on a scale of **1 – 5 where 1 = "Strongly Disagree" and 5 = "Strongly Agree".**

|  | Strongly disagree Strongly agree | | | | | Do not Know |
| --- | --- | --- | --- | --- | --- | --- |
| 1. Gay and lesbian people make me nervous | 1 | 2 | 3 | 4 | 5 | DK |
| 1. Gay and lesbian people deserve any troubles they get | 1 | 2 | 3 | 4 | 5 | DK |
| 1. Homosexuality is acceptable to me | 1 | 2 | 3 | 4 | 5 | DK |
| 1. If I discovered a friend was gay/lesbian I would end the friendship | 1 | 2 | 3 | 4 | 5 | DK |
| 1. I think gay and lesbian people should not work with children | 1 | 2 | 3 | 4 | 5 | DK |
| 1. I sometimes use names like *ah gua, bapok, butch, dyke, gu niang, homo* or *pondan* when talking about gays/lesbians | 1 | 2 | 3 | 4 | 5 | DK |
| 1. I particularly enjoy the company of gays/lesbians | 1 | 2 | 3 | 4 | 5 | DK |
| 1. Marriage between two gay men or lesbian women is acceptable to me | 1 | 2 | 3 | 4 | 5 | DK |
| 1. I sometimes use names like *ah gua, bapok, butch, dyke, gu niang, homo* or *pondan* when talking to people I suspect are gay/lesbian | 1 | 2 | 3 | 4 | 5 | DK |
| 1. It does not matter to me whether my friends are gay/lesbian or straight | 1 | 2 | 3 | 4 | 5 | DK |
| 1. It would not upset me if I learned that a close friend was gay/lesbian | 1 | 2 | 3 | 4 | 5 | DK |
| 1. Homosexuality is immoral | 1 | 2 | 3 | 4 | 5 | DK |
| 1. I make fun of gay/lesbian people | 1 | 2 | 3 | 4 | 5 | DK |
| 1. I feel that you cannot trust a person who is gay/lesbian | 1 | 2 | 3 | 4 | 5 | DK |
| 1. I particularly fear that gay/lesbian people will flirt with me | 1 | 2 | 3 | 4 | 5 | DK |
| 1. Organisations which promote gay/lesbian rights are necessary | 1 | 2 | 3 | 4 | 5 | DK |
| 1. I have damaged property of gay/lesbian people, such as scratching their cars | 1 | 2 | 3 | 4 | 5 | DK |
| 1. I would feel comfortable living with a gay/lesbian person | 1 | 2 | 3 | 4 | 5 | DK |
| 1. I would hit a gay/lesbian person for flirting with me | 1 | 2 | 3 | 4 | 5 | DK |
| 1. Homosexual behaviour should not be against the law | 1 | 2 | 3 | 4 | 5 | DK |
| 1. I avoid gay/lesbian people | 1 | 2 | 3 | 4 | 5 | DK |
| 1. It does not bother me to see two openly gay men/lesbian women together in public | 1 | 2 | 3 | 4 | 5 | DK |
| 1. When I see a gay/lesbian person I think, “What a waste.” | 1 | 2 | 3 | 4 | 5 | DK |
| 1. When I meet someone new I try to find out if he/she is gay/lesbian | 1 | 2 | 3 | 4 | 5 | DK |
| 1. I cannot get along well with people that I suspect are gay/lesbian | 1 | 2 | 3 | 4 | 5 | DK |

Finally, we would like to ask you questions about people who use non-medical/recreational drugs, (e.g. cannabis or heroin). Again, please indicate your level of agreement for the following statements on a scale of **1 – 5 where 1 = "Strongly Disagree" and 5 = "Strongly Agree"** based on your own feelings.

|  | Strongly disagree Strongly agree | | | | |
| --- | --- | --- | --- | --- | --- |
| 1. Using non-medical/recreational drugs is morally wrong | 1 | 2 | 3 | 4 | 5 |
| 1. Non-medical/recreational drug users should go to prison | 1 | 2 | 3 | 4 | 5 |
| 1. Non-medical/recreational drug users are weak minded | 1 | 2 | 3 | 4 | 5 |
| 1. Non-medical/recreational drug users have no future | 1 | 2 | 3 | 4 | 5 |
| 1. Most non-medical/recreational drug users are not well educated | 1 | 2 | 3 | 4 | 5 |
| 1. Non-medical/recreational drug users are dishonest | 1 | 2 | 3 | 4 | 5 |
| 1. Non-medical/recreational drug users make me angry | 1 | 2 | 3 | 4 | 5 |

****Thank you for your time****
