# Supplementary material for: Application of the network scale‐up method to estimate the sizes of key populations for HIV in Singapore using online surveys
Source: J Int AIDS Soc. 2023 Mar 15;26(3):e25973. doi: 10.1002/jia2.25973 (PMC10015632; doi:10.1002/jia2.25973)
Supplement: Supplementary file 2 — Supplement 2: This file contains additional details on the study's methodology and some additional tables of parameters and figures of results that are not the main outcomes but may offer some more insight into the study's findings if required. [file JIA2-26-e25973-s001.docx]

**Application of the network scale-up method to estimate the sizes of key populations for HIV in Singapore using online surveys (supplementary data)**

Table of Contents

[SUPPLEMENTARY MATERIAL FOR THE METHODS SECTION 2](#_Toc104235266)

[**Questionnaire design** 2](#_Toc104235267)

[**Recruitment and participants** 3](#_Toc104235268)

[**Supplementary Figure 1a. Facebook advertisement 1** 3](#_Toc104235269)

[**Supplementary Figure 1b. Facebook advertisement 2** 4](#_Toc104235270)

[**Sampling weights** 4](#_Toc104235271)

[**Analysis** 6](#_Toc104235272)

[**Supplementary Table 1. Data sources and real sizes of the ten reference populations** 6](#_Toc104235273)

[**Supplementary Table 2. Prior distributions of parameters** 8](#_Toc104235274)

[SUPPLEMENTARY MATERIAL FOR THE RESULTS SECTION 8](#_Toc104235275)

[**Supplementary Table 3. Adjusted and unadjusted size estimates of the five key populations from the Bayesian modelling using informative priors** 8](#_Toc104235276)

[**Participants’ acceptability ratings of selected behaviours and populations** 9](#_Toc104235277)

[**Posterior distributions** 18](#_Toc104235281)

# **SUPPLEMENTARY MATERIAL FOR THE METHODS SECTION**

This document provides additional details on our network scale-up study on estimating the sizes of five at-risk populations for HIV/AIDS in Singapore.

## **Questionnaire design**

The original questionnaire was developed following nine in-depth interviews and four focus group discussions with key stakeholders in Singapore’s HIV landscape conducted by some of the authors of the current study. The key populations used in this study arose from some of the major themes and concerns raised in the interviews and discussions [1], which also informed some of the terminology, the incorporation of the social acceptance scores, and the initial choice of reference groups.

The current study used the original questionnaire from the pilot study with some minor changes, motivated by the experience of the pilot and feedback from key stakeholders subsequently. The modified questionnaire underwent pre- and pilot-testing for user acceptability and ease of comprehension. In the current study, additional demographic information on marital status, religion, household income and citizenship status were collected which were not included in the pilot study. For the NSUM component of the questionnaire we used the recommended 10 known populations out of the 20 that were used as reference groups in the pilot study, based on the groups with best out of sample performance in the pilot, as tabulated in Supplementary Table 1. The groups were initially selected based on the qualitative research in the pilot phase with data scientists, with the intent to represent a diverse set of subpopulations that would lead to roughly similar numbers of contacts on aggregate among different groups in the population (for instance, including groups that both younger and older adults might know), and to not favour any particular race or religion.

We also included the transgender population as an additional key population to the four key populations (MCFSW, MSM, FSW and PWID) used in the pilot study. Respondents who indicated that they knew people in the at-risk groups were then asked about the age profiles and ethnicities of these contacts.

## **Recruitment and participants**

Participants for this study were recruited from the Singapore Population Health Studies (SPHS) Online Panel and via targeted Facebook advertisements (ads). The members of the online panel are Singaporeans or Permanent Residents, aged 21 years and above who own personal email accounts, are able to read English, and who use the internet regularly. For recruitment via Facebook, our ads were randomly delivered using Facebook’s algorithms to users that fit our inclusion criteria: Singaporean residents aged 18 years and above. Supplementary figures 1a and 1b below show the Facebook ads that were used to reach our target audience.


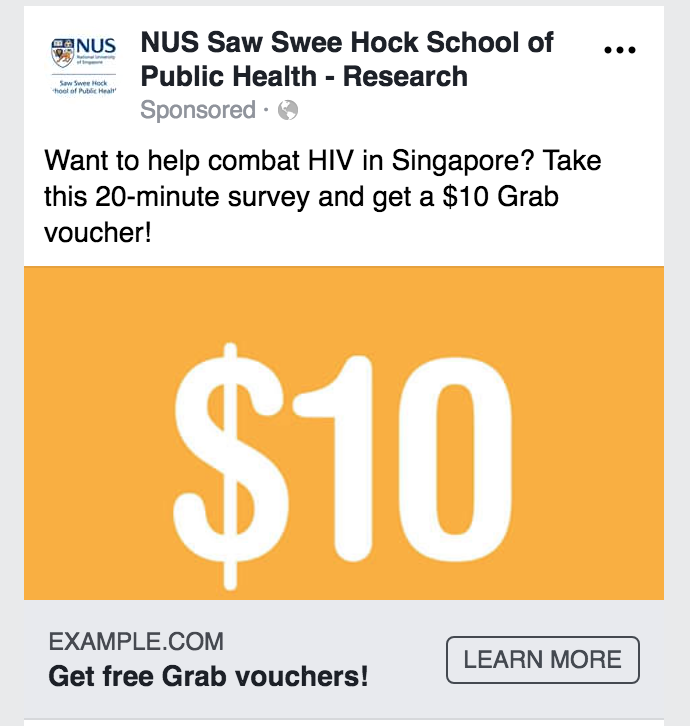


### **Supplementary Figure 1a. Facebook advertisement 1**


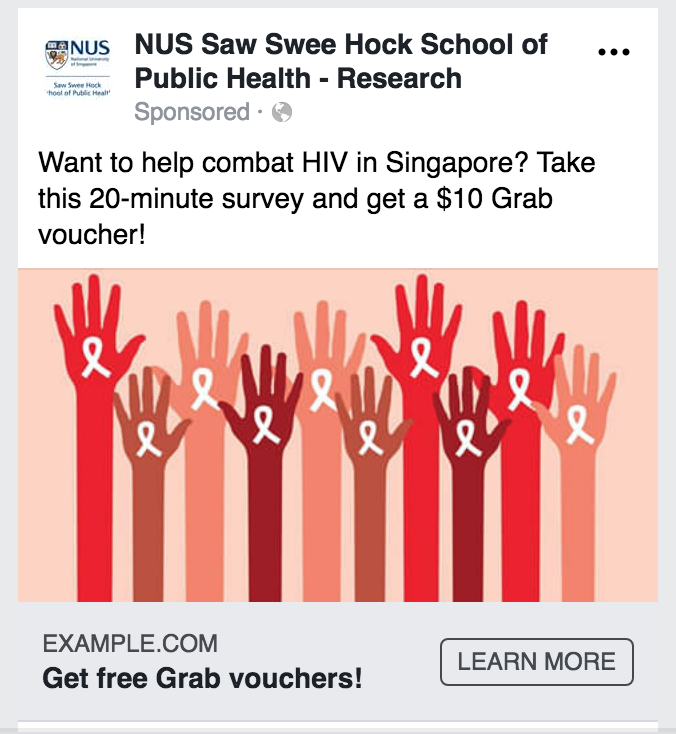


### **Supplementary Figure 1b. Facebook advertisement 2**

Participants who had the ads show up on their Facebook or Instagram timelines would click on the “Learn More” button which would redirect them to our survey website where they could access details about the survey and indicate their consent to be included in our study by clicking on the “Participate” button at the bottom of the online participant information sheet.

## **Sampling weights**

In calculating the weights, we did not split by education for *other* ethnicities owing to small sample sizes in this group. We grouped 18–19-year-olds with 20–29-year-olds to form one age band (18–29-year-olds). For ethnicity, we used the same definitions used for the 2020 population census to reclassify our data. As such, participants who initially selected *others* for their ethnicity and indicated that they were of Indonesian origin were reclassified as *Malays* and those that indicated that they were of Pakistani, Bangladeshi or Sri Lankan origins were reclassified as *Indians*.

## **Analysis**

The following ten reference populations were shortlisted from 20 initial reference populations using leave-one-out validation checks which were performed in the initial pilot study (please see Supplementary Figure 5 in Teo et al 2019) [1]. The validation approach is similar to that recommended by Salganik et al (2011) [2]; in our case we fielded 20 reference groups in the pilot and used the leave-one-out approach to identify the best 10 groups for the full study.

### **Supplementary Table 1. Data sources and real sizes of the ten reference populations**

| **Known Populations** | **Data sources** | **Real size** |
| --- | --- | --- |
| Real estate agents | Council for Estate Agencies | 29 146 |
| Women who had a baby in 2017 | Singapore Statistics | 39 615 |
| Heart attack 2016 | National Registry of Diseases Office | 10 728 |
| Bought a public apartment in 2017 | Data.gov.sg | 28 739 |
| Driving license 2017 | Data.gov.sg | 31 878 |
| Elementary school leaving examination takers in 2018 | Ministry of Education | 39 672 |
| People who attended the annual National Parade in 2018 | The Straits Times | 25 000 |
| Secondary school leaving examination takers in 2018 | Ministry of Education | 26 750 |
| Couples who got married in 2018 | Singapore Statistics | 28 212 |
| Single men older than 50 years | Singapore Statistics | 59 300 |

Legend of table: The real sizes of the 10 reference populations used in our size estimation analysis and their data sources. *Elementary school leaving examination takers, represents students who sat for the elementary school (the equivalent of primary school) leaving examinations in 2018 taken around age 12 in Singapore; National Parade, those who attended Singapore’s annual ticketed Independence Day parade in 2018; Secondary school leaving examination takers, represents students who sat for these examinations in 2018 at age 16 at the end of their secondary school education (the equivalent of junior high school)*.

Two alternative model structures were used, as briefly described in the main manuscript. In both, the same structure was taken for the reference groups, namely the number of individual $i$’s contacts in reference population $j$ was denoted by $N_{ij}^{R}$ and was modelled to have a Poisson distribution with mean $\lambda\alpha_{i}S_{j}^{R}$. The three components of the mean are a random effect, $\alpha_{i}$, to account for differences from person to person in their total network size; the size of the reference population, $S_{j}^{R}$, which is known; and a scaling factor $\lambda$, which represents the proportion of the population that falls into a typical person’s social network. The size of a typical social network can be derived from the product of $\lambda$ and the total size of the country.

The two model structures differ in how the hidden populations are accounted for. In the *unadjusted* model, the same structure is taken as with the reference groups, i.e. $N_{ij}^{H}$ is taken to be Poisson with mean $\lambda\alpha_{i}S_{j}^{H}$, only this time the hidden population size $S_{j}^{H}$ is unknown and hence an estimand in the analysis. In the *adjusted* model, the mean is taken instead to be $\lambda\alpha_{i}\tau_{ij}S_{j}^{H}$ where $\tau_{ij}=\exp\left\{ \upsilon_{j}\left( x_{ij}-U_{j} \right) \right\}$, and $x_{ij}$ is the social acceptability score that $i$ reports towards group $j$, with $U_{j}$ the maximum value that can be held. The $\nu_{j}$ parameter therefore allows for transmission error to occur for those who report lower acceptability scores; if $\nu_{j}=0$ then no transmission error is present. Differences in reported number of contacts in the hidden group between participants with different acceptability scores is what allows $\nu_{j}$ to be estimated.

A complication to account for is the need to address over- or under-sampling of some demographic segments in the analysis. In Bayesian analyses it is not straightforward to incorporate sampling weights within the likelihood calculations, so instead we took an approach inspired by Rubin’s multiple imputation method [2]. We repeatedly resampled the data using posthoc sampling weights to select an unweighted sample of the same size and hence information content as the original data. For each resampled dataset, we derived a sample of the posterior distribution of the unknown parameters using Markov chain Monte Carlo sampling. This was repeated $M=100$ times to derive 100 samples of size $m=50 000$ each. As the posteriors of the hidden population sizes were slightly right skewed, these were first log transformed, and the posterior mean and variance of the log population sizes calculated for resample $r$, using the notation $\mu_{jr}$ and $\sigma_{jr}^{2}$ for subpopulation $j$. The overall mean and variance averaging across the 100 resamples were then derived using Rubin’s formula as implemented using Amelia II package [3] by Honaker et al to pool the estimates accounting for the variance within and between the resamples.

From this, a point estimate and 95% uncertainty interval were derived, which were then exponentiated to the natural scale. These are the estimates reported in the paper. The 100 estimates are presented in supplementary figures 6–9 in this document.

The posterior distributions were derived using prior distributions tabulated in supplementary table 2.

### **Supplementary Table 2. Prior distributions of parameters**

| Model | Parameter | Notation | Prior Density |
| --- | --- | --- | --- |
| Basic Bayesian NSUM model (*unadjusted*) | Social acceptability parameter | $\alpha_{i}$ | $\alpha_{i}$ ~ logN(0,$\xi$) |
|  | Social acceptability dispersion | $\xi$ | $\xi$ ~ U(0, 10) |
|  | Scale parameter | $\lambda$ | $\lambda$ ~ U(0, 10) |
|  | Known population | $s_{j}^{K}$ | Data |
|  | Hidden population | $s_{l}^{H}$ | $s_{l}^{H}$ ~ U(0, 2500 000) |
| Transmission error model (*adjusted*) | Social acceptability parameter | $\nu_{j}$ | $\nu_{j}$ ~ N(0, $\sigma_{\nu}$) |
|  | Social acceptability dispersion | $\sigma_{\nu}$ | $\sigma_{\nu}$ ~ Gamma(1, 0.1) |

# **SUPPLEMENTARY MATERIAL FOR THE RESULTS SECTION**

### **Supplementary Table 3. Adjusted and unadjusted size estimates of the five key populations from the Bayesian modelling using informative priors**

| **Population** | **Unadjusted size (IP)**^¶^ | | **95% CI*** | **Adjusted size (IP)**^†^ | **95% CI*** | |
| --- | --- | --- | --- | --- | --- | --- |
| **MCFSW** | 29 900 | | (27 000 – 33 100) | 76 600 | (64 300 – 91 400) | |
| **Transgender** | 9 180 | | (7 980 – 10 600) | 18 000 | (14 000 – 23 300) | |
| **MSM** | 44 000 | | (40 100 – 48 300) | 138 000 | (120 000 – 159 000) | |
| **FSW** | 5 120 | | (3 780 – 6 930) | 7 900 | (3 880 – 16 100) | |
| **PWID** | 1 800 | | (1 410 – 2 310) | 3 260 | (1 440 – 7 410) | |
| **Personal network** | | 142 | (134 – 150) | 142 | (134 – 150) |  |

Legend of table: * 95% CI represents the credible interval of the mean estimates. ¶ Unadjusted size estimates were derived accounting for barrier effects but not transmission error. † The adjusted size estimates were derived after accounting for transmission error and barrier effects. IP, informative priors; FSW, female sex workers; PWID, people who inject drugs; MCFSW, male clients of female sex workers; MSM, men who have sex with men.

## **Participants’ acceptability ratings of selected behaviours and populations**

Participants were asked to rate the acceptability of selected behaviours and populations on a 10-point Likert scale from completely unacceptable on one end to completely acceptable on the other end. Supplementary figures 2 to 5 show these results in graphical form. Overall, participants were generally unaccepting of the behaviours of members of the key populations, especially injecting illicit drugs (supplementary figure 2). Of all the high-risk groups, the population seems to be more accepting of transgender people—in the survey, the question was phrased as how acceptable or unacceptable would they rate having a sex change.

We also analysed the results by sex (supplementary figure 3), age (supplementary figure 4) and ethnicity (supplementary figure 5). Between sexes, males are more accepting of the behaviours of members of the key populations compared to females. Between ages, we found that younger people generally tend to rate the behaviours of members in the high-risk groups more favourably compared to older people. Between ethnicities, Indians were more accepting of the behaviours of those in the high-risk groups than were Malays and Chinese.


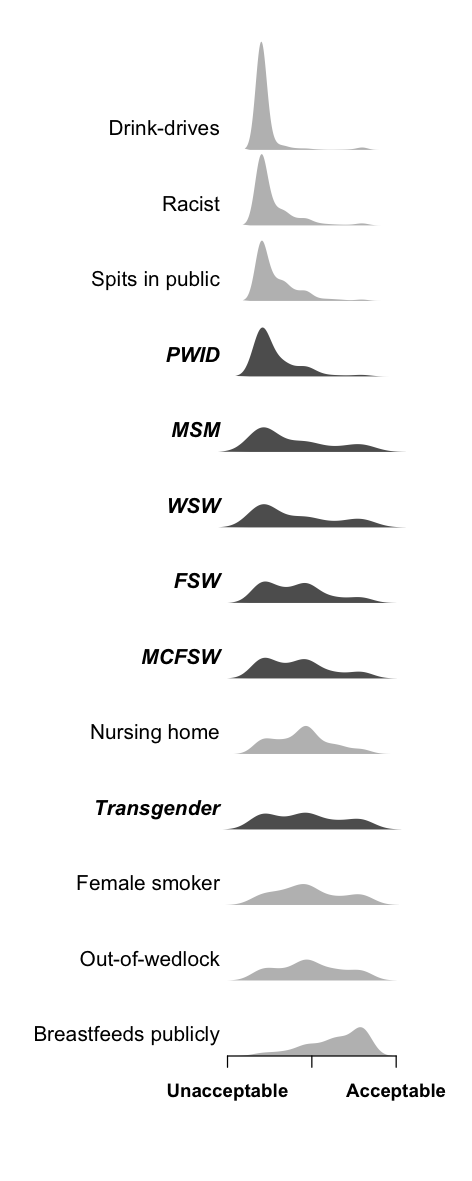


**Supplementary Figure 2. Overall distribution of participants’ social acceptability ratings***. PWID, people who inject drugs; MSM, men who have sex with men; FSW, female sex workers; WSW, women who have sex with women; MCFSW, male clients of female sex workers.*

**Supplementary Table 4. Overall means and standard deviations of participants’ social acceptability ratings**

| **Behaviour** | **Mean (SD)** |
| --- | --- |
| Someone driving while drunk (Drink-drives) | 1.4 (1.5) |
| Someone who makes racist comments (Racist) | 2.0 (1.8) |
| Someone who spits in public (Spits in public) | 2.3 (1.8) |
| Someone who takes non-medical drugs by injections (PWID) | 2.3 (2.0) |
| A man who has sex with another man (MSM) | 3.8 (3.3) |
| A woman who has sex with another woman (WSW) | 4.0 (3.3) |
| A woman who sells sex (FSW) | 4.1 (2.8) |
| A man who pays for sex with a woman (MCFSW) | 4.2 (2.9) |
| Someone who puts their parents in a nursing home (Nursing home) | 4.6 (2.5) |
| A person who had a sex change (Transgender) | 5.0 (3.2) |
| A woman who smokes cigarettes (Female smoker) | 5.2 (2.9) |
| A woman who is pregnant before marriage (Out-of-wedlock) | 5.3 (2.9) |
| A woman who breastfeeds publicly (Breastfeeds publicly) | 7.6 (2.6) |


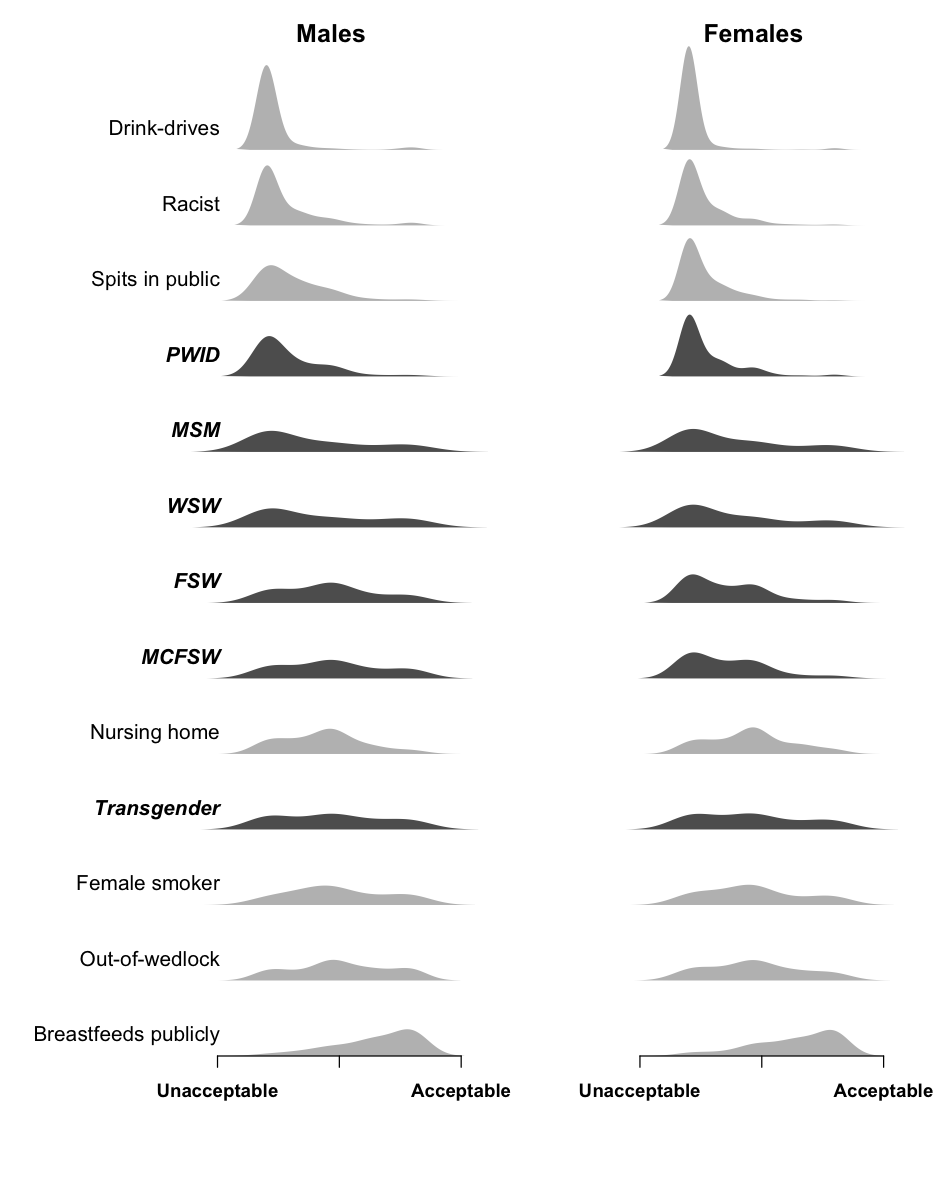


### **Supplementary Figure 3. Distribution of participants’ social acceptability ratings by sex.** *PWID, people who inject drugs; MSM, men who have sex with men; FSW, female sex workers; WSW, women who have sex with women; MCFSW, male clients of female sex workers.*

**Supplementary Table 5. Means and standard deviations of participants’ social acceptability ratings by sex**

| **Behaviour** | **Mean (SD)** | |
| --- | --- | --- |
|  | **Males** | **Females** |
| Someone driving while drunk (Drink-drives) | 1.5 (1.7) | 1.3 (1.3) |
| Someone who makes racist comments (Racist) | 2.2 (2.0) | 1.9 (1.6) |
| Someone who spits in public (Spits in public) | 2.6 (2.0) | 2.0 (1.6) |
| Someone who takes non-medical drugs by injections (PWID) | 2.4 (2.0) | 2.1 (1.9) |
| A man who has sex with another man (MSM) | 3.9 (3.3) | 3.7 (3.2) |
| A woman who has sex with another woman (WSW) | 4.3 (3.4) | 3.7 (3.2) |
| A woman who sells sex (FSW) | 4.9 (2.9) | 3.3 (2.4) |
| A man who pays for sex with a woman (MCFSW) | 5.1 (3.0) | 3.4 (2.4) |
| Someone who puts their parents in a nursing home (Nursing home) | 4.5 (2.5) | 4.8 (2.5) |
| A person who had a sex change (Transgender) | 5.1 (3.2) | 4.9 (3.1) |
| A woman who smokes cigarettes (Female smoker) | 5.4 (2.9) | 5.0 (2.9) |
| A woman who is pregnant before marriage (Out-of-wedlock) | 5.6 (2.9) | 5.1 (2.8) |
| A woman who breastfeeds publicly (Breastfeeds publicly) | 7.7 (2.5) | 7.5 (2.6) |


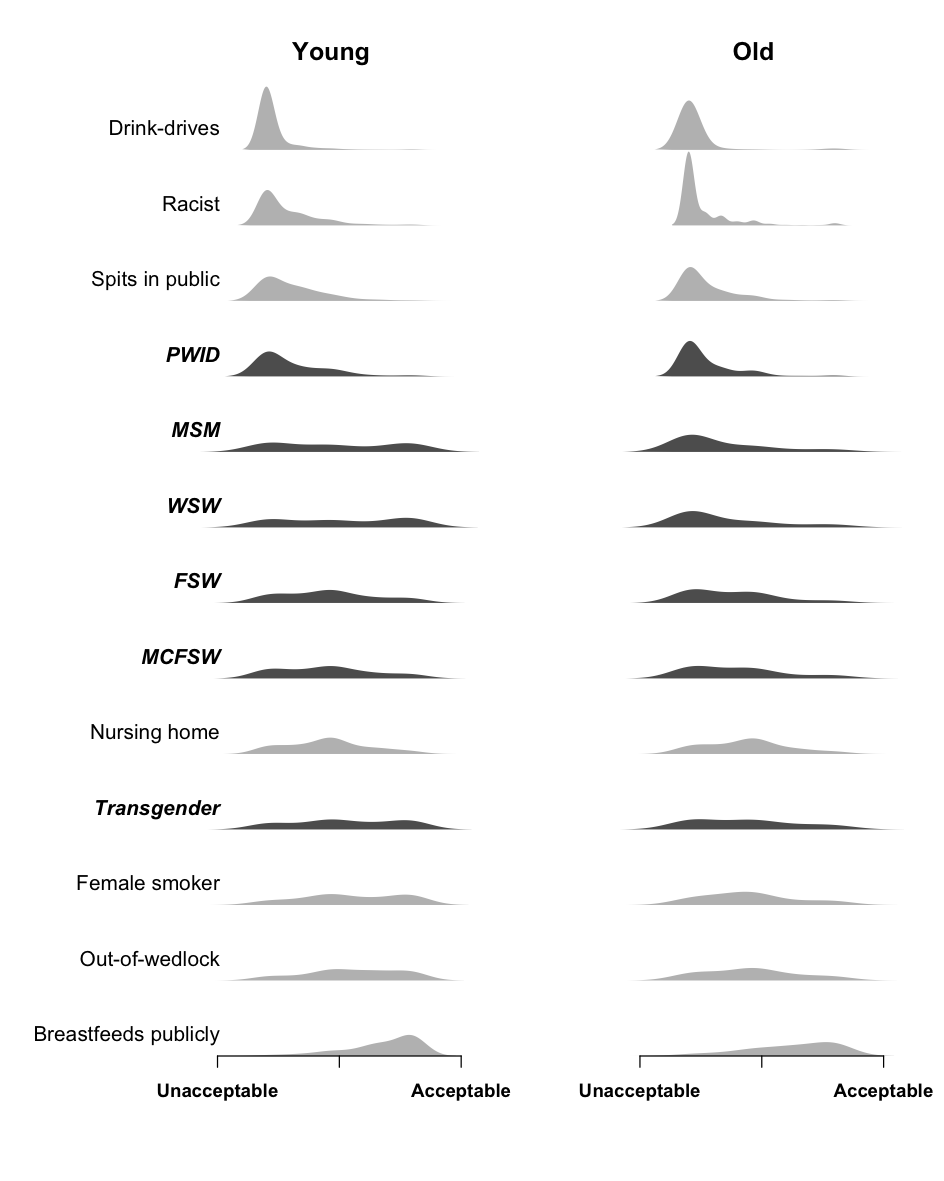


### **Supplementary Figure 4. Distribution of participants’ social acceptability ratings by age.** *PWID, people who inject drugs; MSM, men who have sex with men; FSW, female sex workers; WSW, women who have sex with women; MCFSW, male clients of female sex workers.*

**Supplementary Table 6. Means and standard deviations of participants’ social acceptability ratings by age**

| **Behaviour** | **Mean (SD)** | |
| --- | --- | --- |
|  | **Young** | **Old** |
| Someone driving while drunk (Drink-drives) | 1.4 (1.3) | 1.4 (1.6) |
| Someone who makes racist comments (Racist) | 2.3 (1.9) | 1.9 (1.8) |
| Someone who spits in public (Spits in public) | 2.6 (1.9) | 2.2 (1.8) |
| Someone who takes non-medical drugs by injections (PWID) | 2.6 (2.1) | 2.1 (1.9) |
| A man who has sex with another man (MSM) | 5.3 (3.5) | 3.1 (2.9) |
| A woman who has sex with another woman (WSW) | 5.7 (3.5) | 3.3 (3.0) |
| A woman who sells sex (FSW) | 4.9 (2.8) | 3.7 (2.7) |
| A man who pays for sex with a woman (MCFSW) | 4.7 (2.8) | 4.0 (2.8) |
| Someone who puts their parents in a nursing home (Nursing home) | 4.8 (2.5) | 4.6 (2.5) |
| A person who had a sex change (Transgender) | 5.9 (3.2) | 4.5 (3.1) |
| A woman who smokes cigarettes (Female smoker) | 6.2 (3.0) | 4.7 (2.7) |
| A woman who is pregnant before marriage (Out-of-wedlock) | 6.3 (2.8) | 4.9 (2.8) |
| A woman who breastfeeds publicly (Breastfeeds publicly) | 8.1 (2.3) | 7.3 (2.6) |


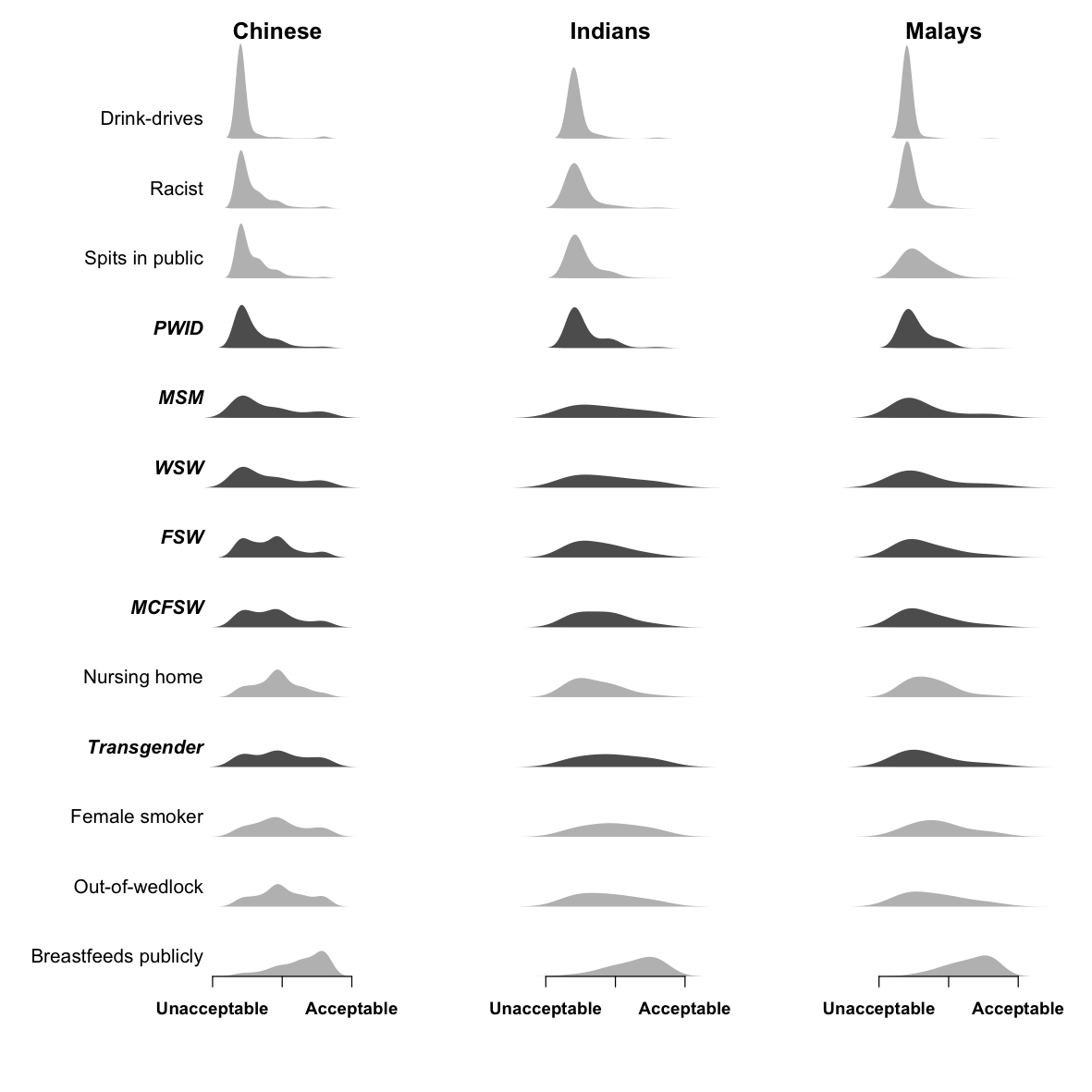


### **Supplementary Figure 5. Distribution of participants’ social acceptability ratings by ethnicity.** *PWID, people who inject drugs; MSM, men who have sex with men; FSW, female sex workers; WSW, women who have sex with women; MCFSW, male clients of female sex workers.*

**Supplementary Table 7. Means and standard deviations of participants’ social acceptability ratings by ethnicity**

| **Behaviour** | **Mean (SD)** | | |
| --- | --- | --- | --- |
|  | **Chinese** | **Indians** | **Malays** |
| Someone driving while drunk (Drink-drives) | 1.4 (1.5) | 1.4 (1.3) | 1.1 (0.7) |
| Someone who makes racist comments (Racist) | 2.2 (1.9) | 1.7 (1.7) | 1.3 (0.9) |
| Someone who spits in public (Spits in public) | 2.3 (1.9) | 1.8 (1.5) | 2.3 (1.6) |
| Someone who takes non-medical drugs by injections (PWID) | 2.3 (2.0) | 2.1 (1.9) | 2.0 (1.6) |
| A man who has sex with another man (MSM) | 3.7 (3.2) | 4.4 (3.4) | 2.9 (3.1) |
| A woman who has sex with another woman (WSW) | 4.0 (3.3) | 4.5 (3.4) | 3.1 (3.2) |
| A woman who sells sex (FSW) | 4.2 (2.7) | 3.7 (2.7) | 3.1 (2.8) |
| A man who pays for sex with a woman (MCFSW) | 4.4 (2.8) | 4.0 (2.7) | 3.1 (2.7) |
| Someone who puts their parents in a nursing home (Nursing home) | 4.9 (2.4) | 3.4 (2.5) | 3.4 (2.3) |
| A person who had a sex change (Transgender) | 5.1 (3.1) | 5.2 (3.2) | 3.3 (3.0) |
| A woman who smokes cigarettes (Female smoker) | 5.2 (2.8) | 5.3 (3.1) | 4.6 (2.8) |
| A woman who is pregnant before marriage (Out-of-wedlock) | 5.5 (2.7) | 4.6 (3.1) | 4.0 (3.1) |
| A woman who breastfeeds publicly (Breastfeeds publicly) | 7.5 (2.6) | 7.6 (2.6) | 7.8 (2.4) |

## **Posterior distributions**

The following plots show the adjusted and unadjusted posterior distributions of each of the five key populations for the estimates when both non-informative and informative priors are used.

**Supplementary figures 6a, 6b, 6c, 6d, 6e** are plots of the adjusted estimates of the sizes of five key populations using non-informative priors: male clients of female sex workers, transgender individuals, men who have sex with men, female sex workers and people who inject drugs. For each bootstrap sample, we plotted the posterior mean (black dot) and 95% credible interval (attached vertical bar) for the population size estimate. In addition, we computed the pooled estimates by using Rubin’s method and plotted the mean and 95% confidence interval, represented by the thicker highlighted bar at the rightmost side of each figure.


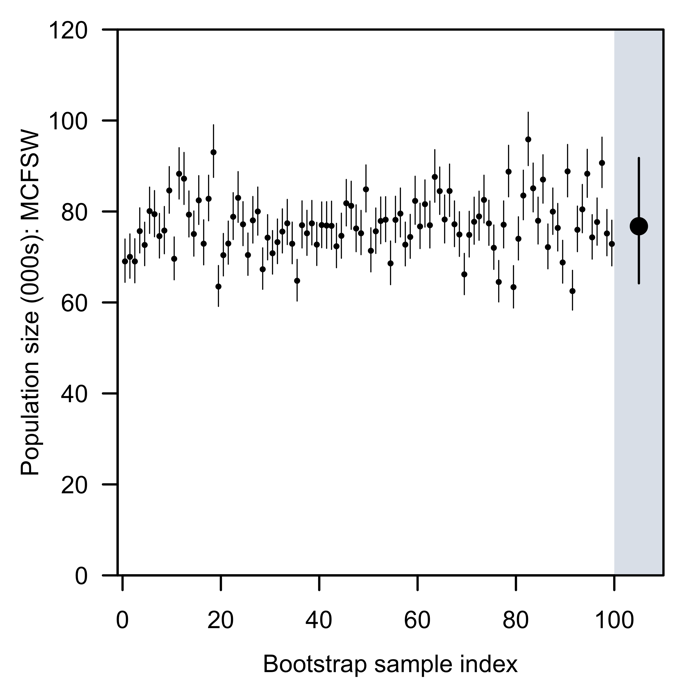


**Supplementary Figure 6a. Posterior means and 95% CIs of the adjusted estimates of MCFSW when non-informative priors are used**


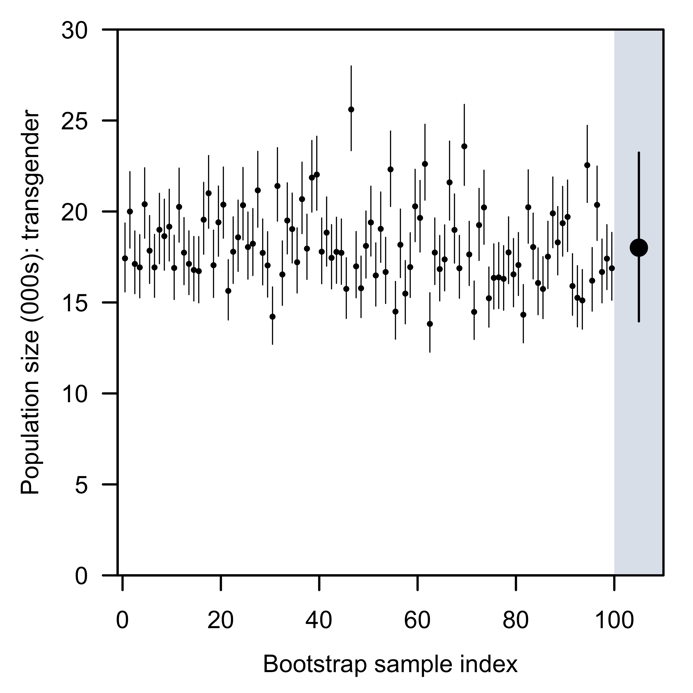


**Supplementary Figure 6b. Posterior means and 95% CIs of the adjusted estimates of transgender persons when non-informative priors are used**


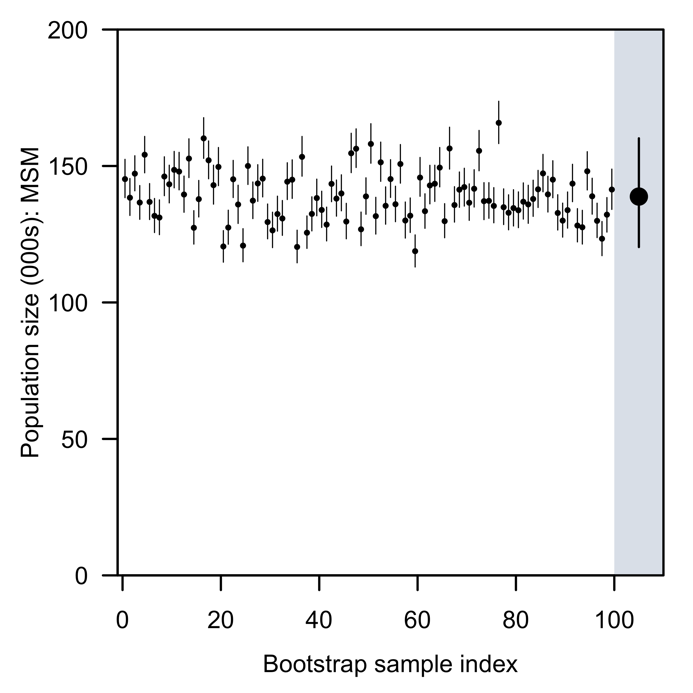


**Supplementary Figure 6c. Posterior means and 95% CIs of the adjusted estimates of MSM when non-informative priors are used**


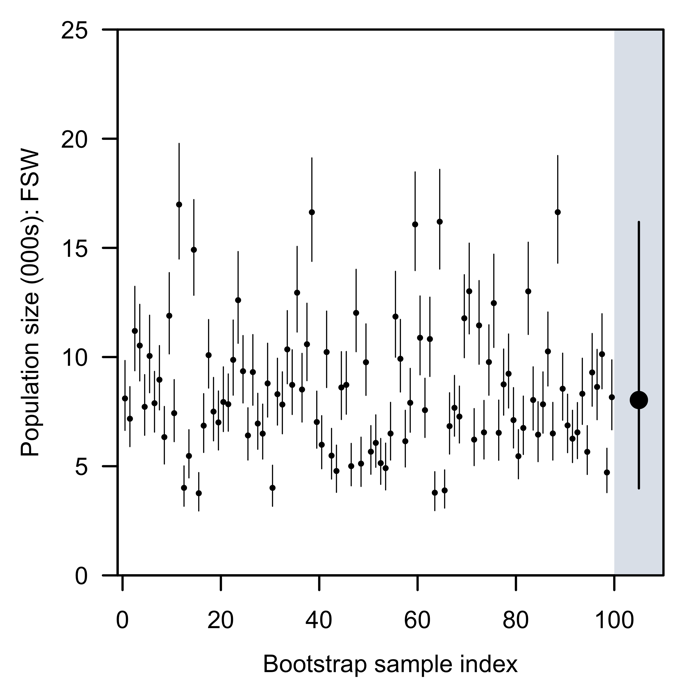


**Supplementary Figure 6d. Posterior means and 95% CIs of the adjusted estimates of FSW when non-informative priors are used**


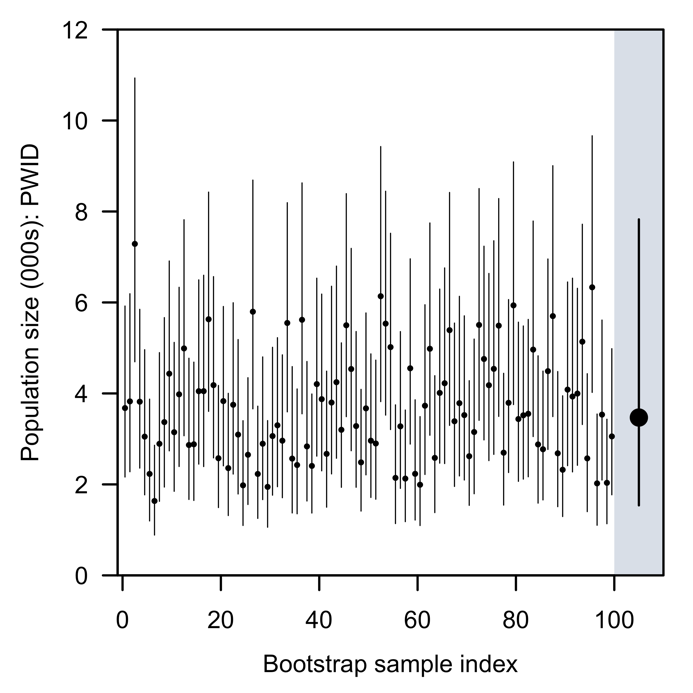


**Supplementary Figure 6e. Posterior means and 95% CIs of the adjusted estimates of PWID when non-informative priors are used**

**Supplementary figures 7a, 7b, 7c, 7d, 7e** are plots of the adjusted estimates of the sizes of five key populations using informative priors: male clients of female sex workers, transgender individuals, men who have sex with men, female sex workers and people who inject drugs. For each bootstrap sample, we plotted the posterior mean (black dot) and 95% credible interval (attached vertical bar) for the population size estimate. In addition, we computed the pooled estimates by using Rubin’s method and plotted the mean and 95% confidence interval, represented by the thicker highlighted bar at the rightmost side of each figure.


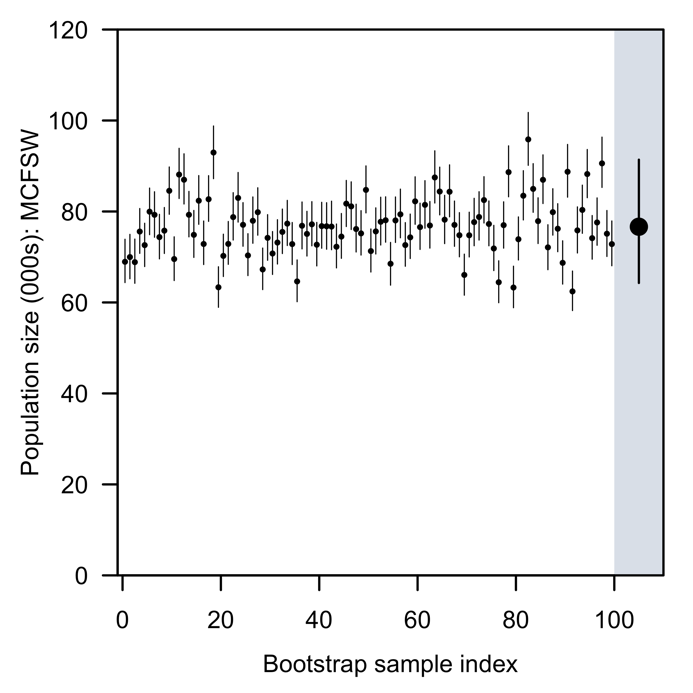


**Supplementary Figure 7a. Posterior means and 95% CIs of the adjusted estimates of MCFSW when informative priors are used**


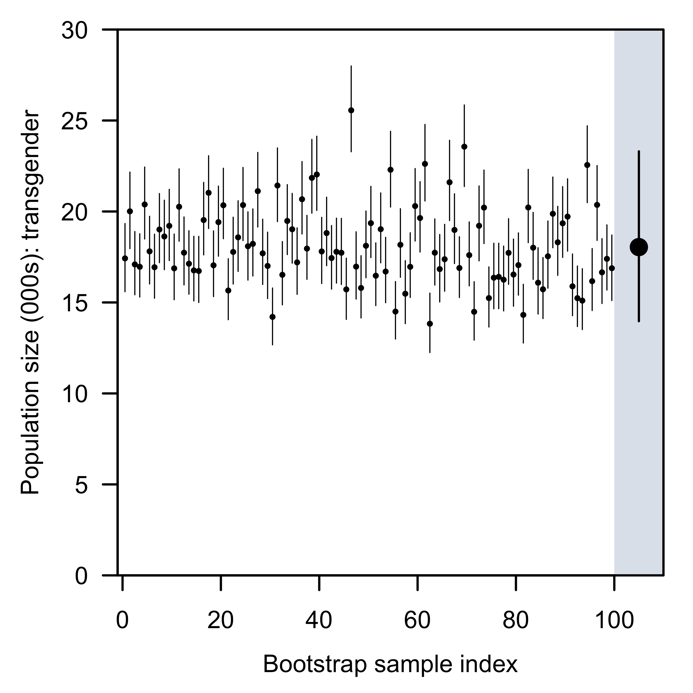


**Supplementary Figure 7b. Posterior means and 95% CIs of the adjusted estimates of transgender persons when informative priors are used**


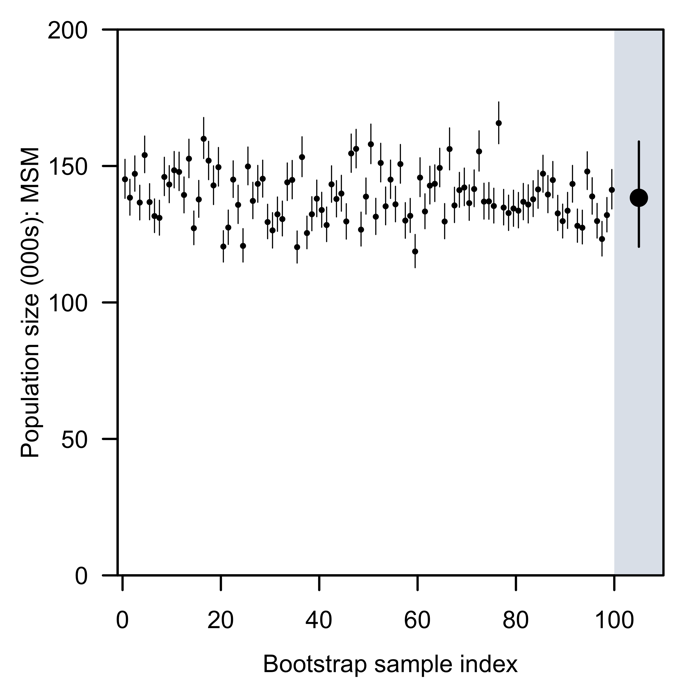


**Supplementary Figure 7c. Posterior means and 95% CIs of the adjusted estimates of MSM when informative priors are used**


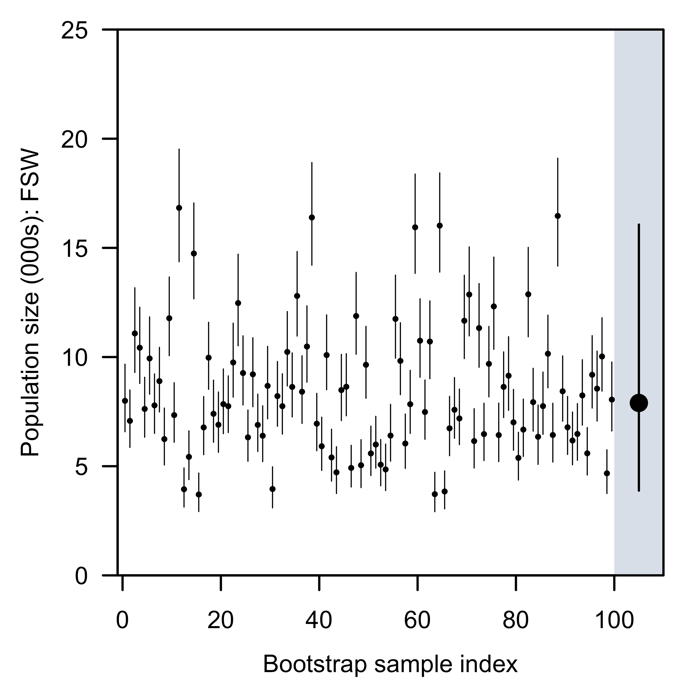


**Supplementary Figure 7d. Posterior means and 95% CIs of the adjusted estimates of FSW when informative priors are used**


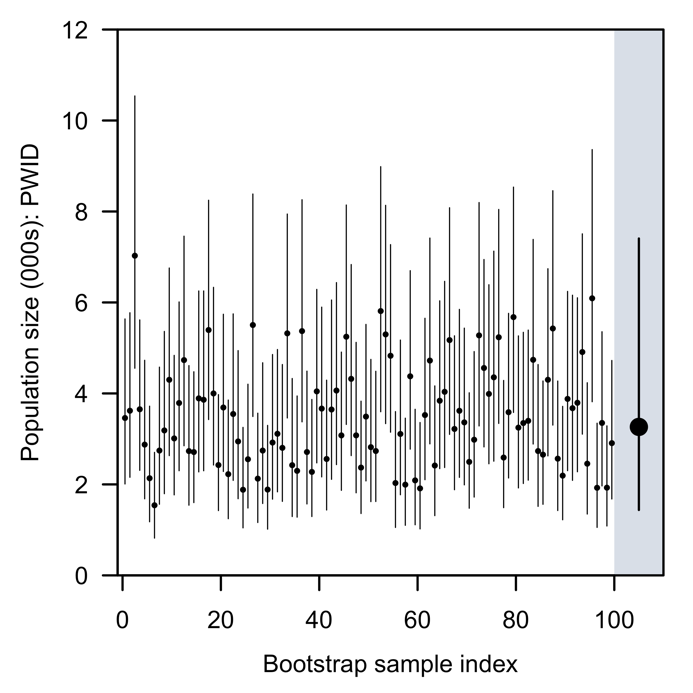


**Supplementary Figure 7e. Posterior means and 95% CIs of the adjusted estimates of PWID when informative priors are used**

**Supplementary figures 8a, 8b, 8c, 8d, 8e** are plots of the unadjusted estimates of the sizes of five key populations using non-informative priors: male clients of female sex workers, transgender individuals, men who have sex with men, female sex workers and people who inject drugs. For each bootstrap sample, we plotted the posterior mean (black dot) and 95% credible interval (attached vertical bar) for the population size estimate. In addition, we computed the pooled estimates by using Rubin’s method and plotted the mean and 95% confidence interval, represented by the thicker highlighted bar at the rightmost side of each figure.


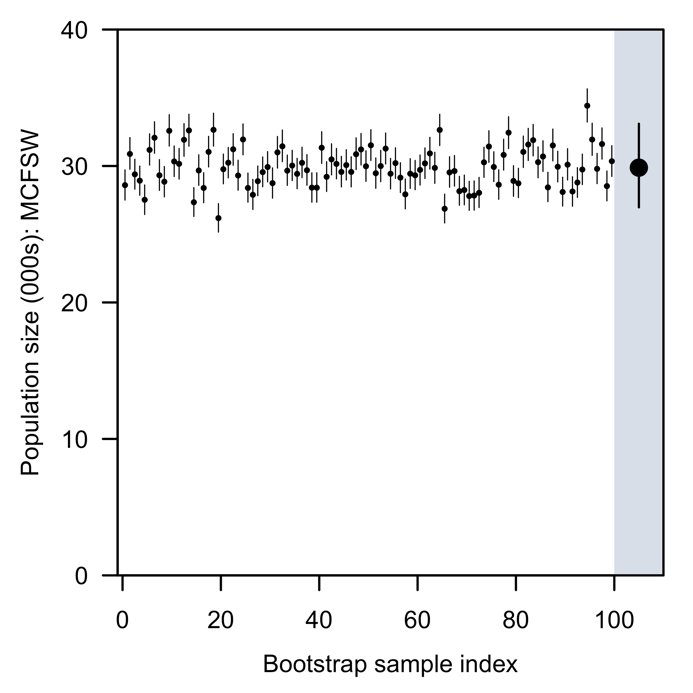


**Supplementary Figure 8a. Posterior means and 95% CIs of the unadjusted estimates of MCFSW when non-informative priors are used**


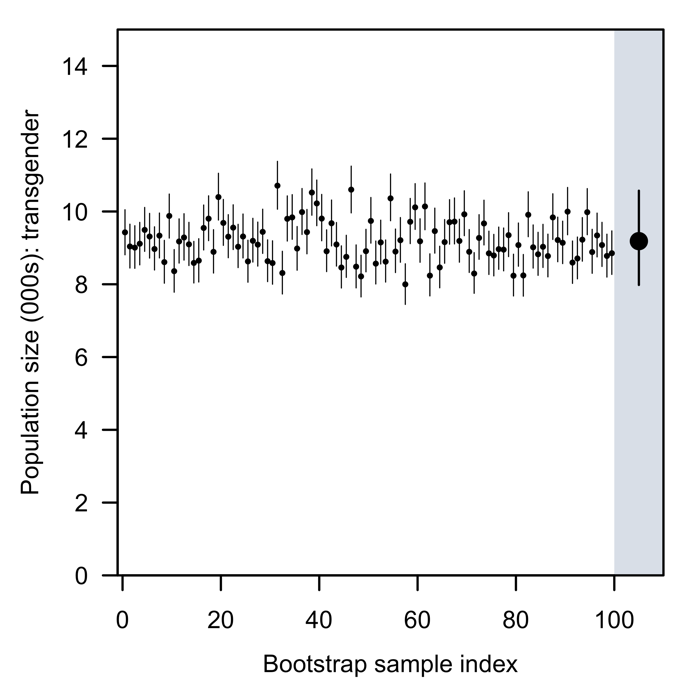


**Supplementary Figure 8b. Posterior means and 95% CIs of the unadjusted estimates of transgender persons when non-informative priors are used**


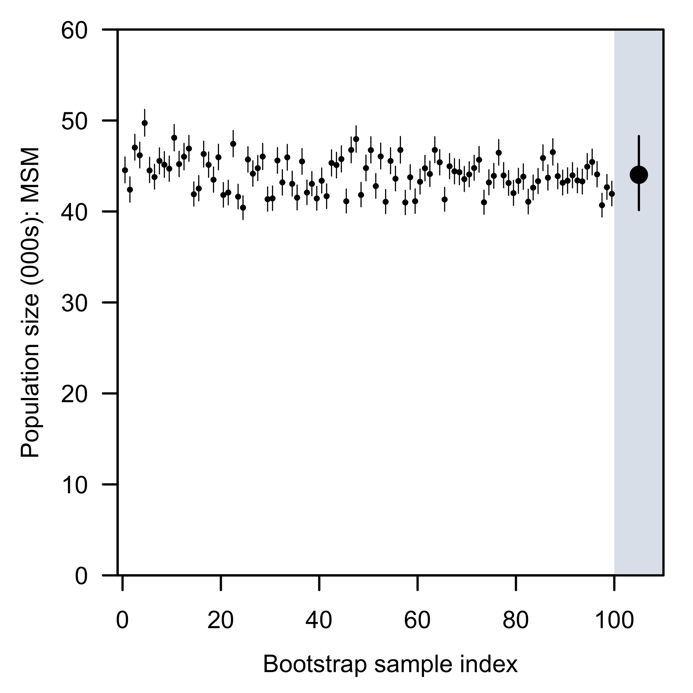


**Supplementary Figure 8c. Posterior means and 95% CIs of the unadjusted estimates of MSM when non-informative priors are used**


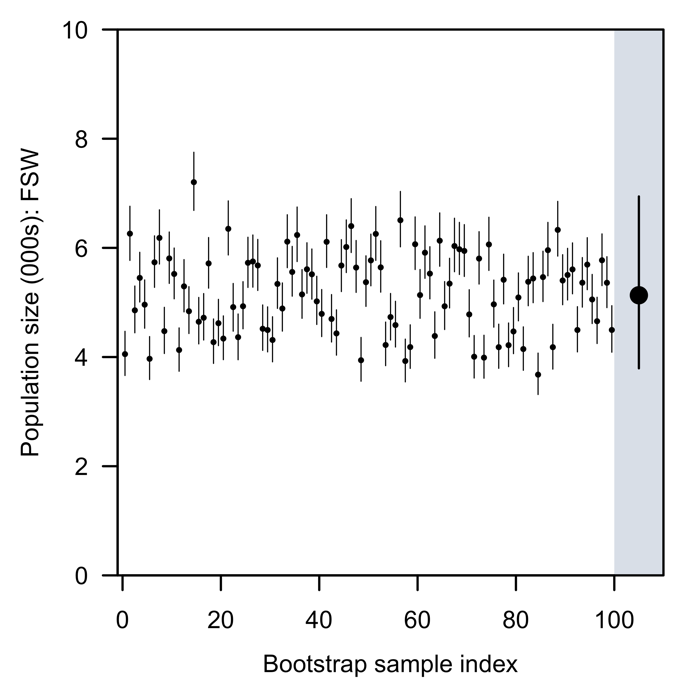


**Supplementary Figure 8d. Posterior means and 95% CIs of the unadjusted estimates of FSW when non-informative priors are used**


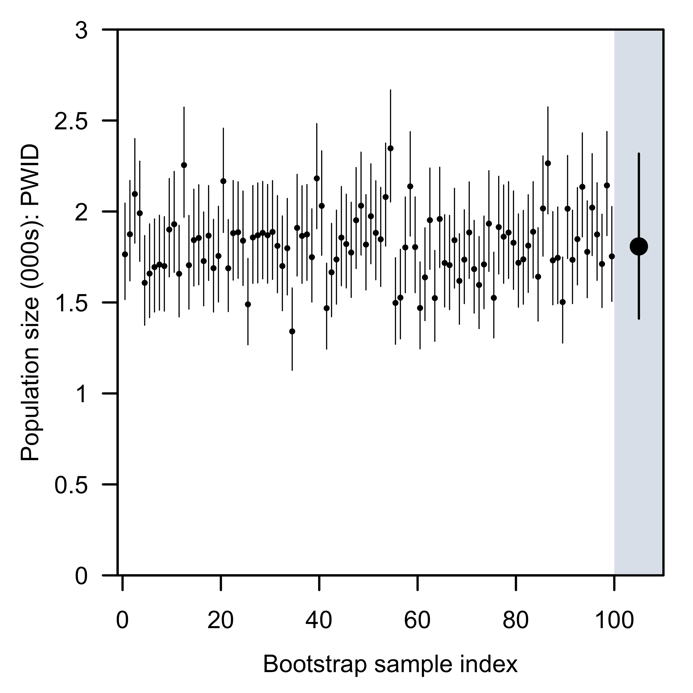


**Supplementary Figure 8e. Posterior means and 95% CIs of the unadjusted estimates of PWID when non-informative priors are used**

**Supplementary figures 9a, 9b, 9c, 9d, 9e** are plots of the unadjusted estimates of the sizes of five key populations using informative priors: male clients of female sex workers, transgender individuals, men who have sex with men, female sex workers and people who inject drugs. For each bootstrap sample, we plotted the posterior mean (black dot) and 95% credible interval (attached vertical bar) for the population size estimate. In addition, we computed the pooled estimates by using Rubin’s method and plotted the mean and 95% confidence interval, represented by the thicker highlighted bar at the rightmost side of each figure.


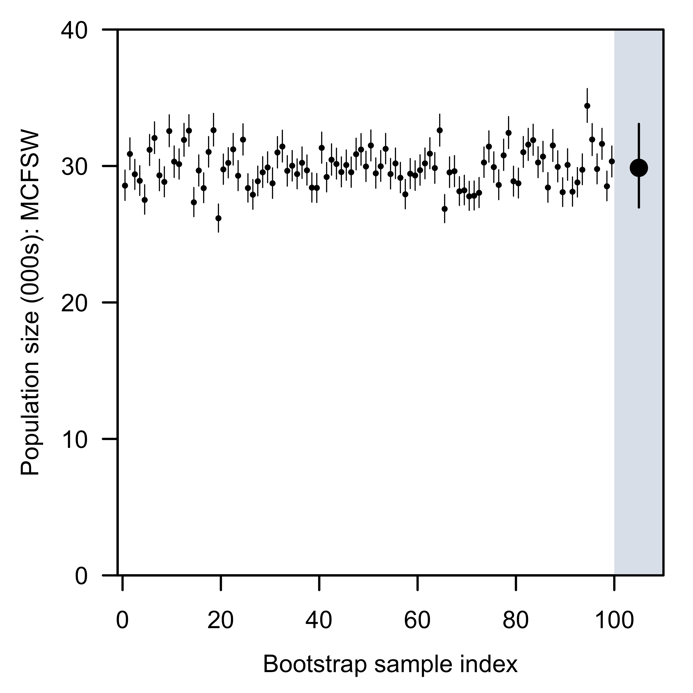


**Supplementary Figure 9a. Posterior means and 95% CIs of the unadjusted estimates of MCFSW when informative priors are used**


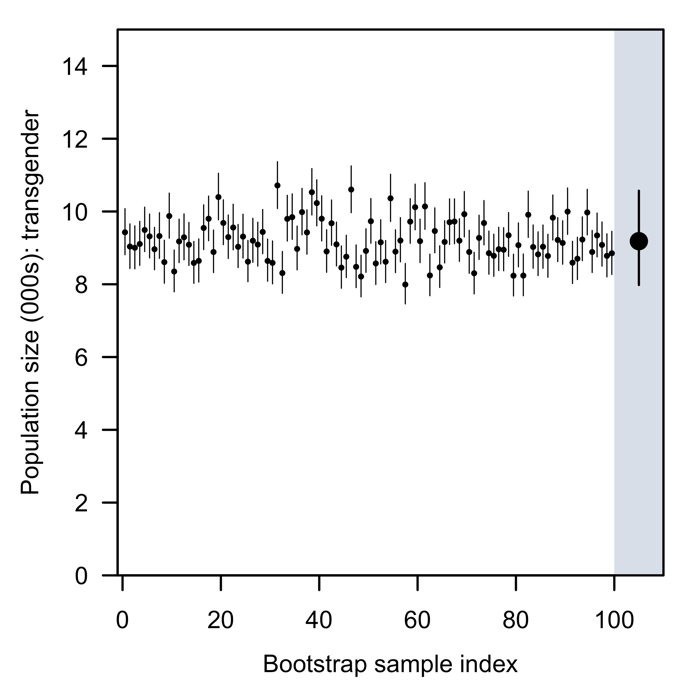


**Supplementary Figure 9b. Posterior means and 95% CIs of the unadjusted estimates of transgender persons when informative priors are used**


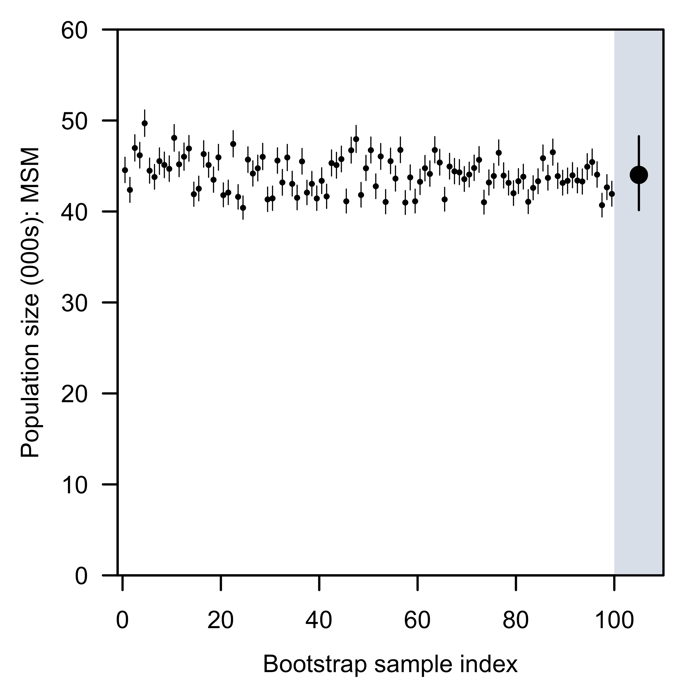


**Supplementary Figure 9c. Posterior means and 95% CIs of the unadjusted estimates of MSM when informative priors are used**


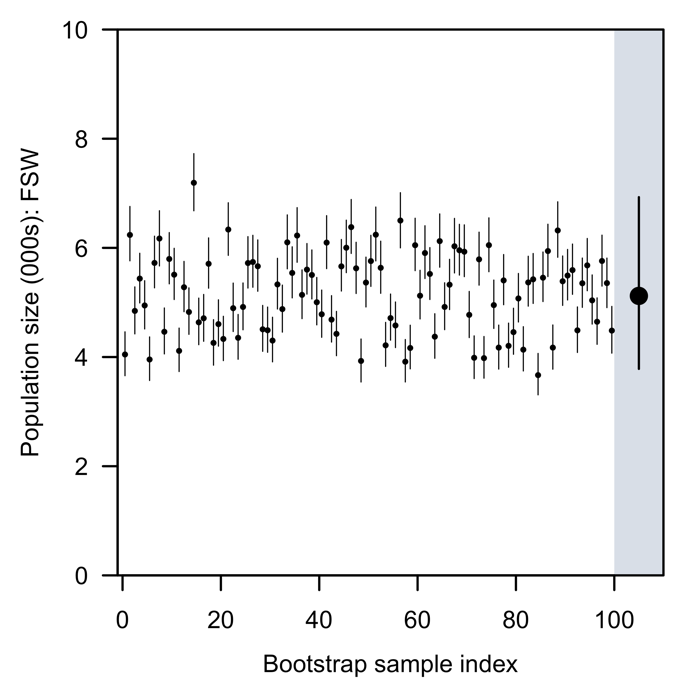


**Supplementary Figure 9d. Posterior means and 95% CIs of the unadjusted estimates of FSW when informative priors are used**


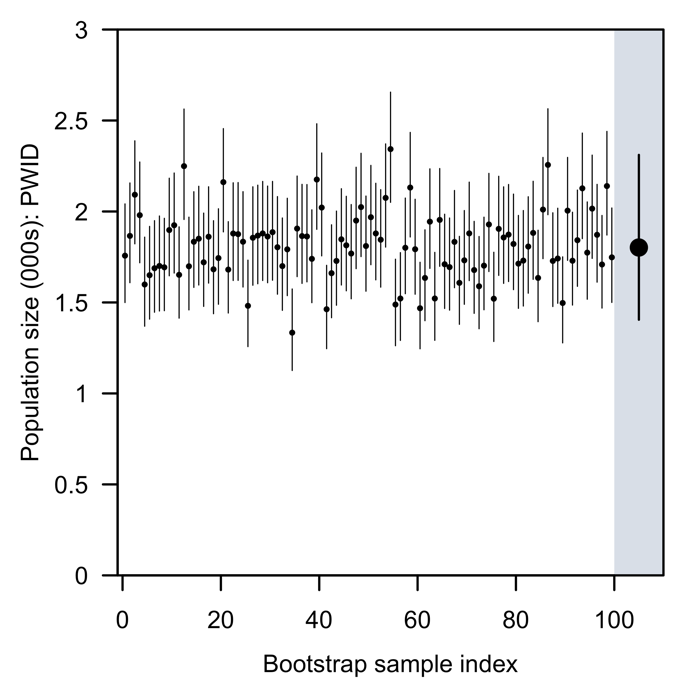


**Supplementary Figure 9e. Posterior means and 95% CIs of the unadjusted estimates of PWID when informative priors are used**

**References**

1. Teo AKJ, Prem K, Chen MIC, Roellin A, Wong ML, La HH, et al. Estimating the size of key populations for HIV in Singapore using the network scale-up method. Sex Transm Infect. 2019 May 8;sextrans-2018-053747.

2. Salganik MJ, Fazito D, Bertoni N, Abdo AH, Mello MB, Bastos FI. Assessing Network Scale-up Estimates for Groups Most at Risk of HIV/AIDS: Evidence From a Multiple-Method Study of Heavy Drug Users in Curitiba, Brazil. Am J Epidemiol. 2011 Nov 15;174(10):1190–6.

3. Rubin DB. Multiple Imputation for Nonresponse in Surveys. New Jersey: John Wiley & Sons, Inc.; 1987.

4. Honaker J, King G, Blackwell M. Amelia II: A Program for Missing Data. Journal of Statistical Software. 2011 Dec 12;45:1–47.
